# Structural Catalytic Core of the Members of the Superfamily of Acid Proteases

**DOI:** 10.3390/molecules29153451

**Published:** 2024-07-23

**Authors:** Alexander I. Denesyuk, Konstantin Denessiouk, Mark S. Johnson, Vladimir N. Uversky

**Affiliations:** 1 Structural Bioinformatics Laboratory, Biochemistry, InFLAMES Research Flagship Center, Faculty of Science and Engineering, Åbo Akademi University, 20520 Turku, Finland; kdenessi@abo.fi (K.D.); mark.s.johnson@abo.fi (M.S.J.); 2 Department of Molecular Medicine and USF Health Byrd Alzheimer’s Research Institute, Morsani College of Medicine, University of South Florida, Tampa, FL 33612, USA

**Keywords:** pepsin, retropepsin, Ddi1, Lpg0085, acid protease, three-dimensional structure, active site, catalytic aspartate

## Abstract

The superfamily of acid proteases has two catalytic aspartates for proteolysis of their peptide substrates. Here, we show a minimal structural scaffold, the structural catalytic core (SCC), which is conserved within each family of acid proteases, but varies between families, and thus can serve as a structural marker of four individual protease families. The SCC is a dimer of several structural blocks, such as the DD-link, D-loop, and G-loop, around two catalytic aspartates in each protease subunit or an individual chain. A dimer made of two (D-loop + DD-link) structural elements makes a DD-zone, and the D-loop + G-loop combination makes a psi-loop. These structural markers are useful for protein comparison, structure identification, protein family separation, and protein engineering.

## 1. Introduction

Earlier, we described structural catalytic cores in many serine and cysteine proteases and showed the presence of unique structure/functional environments, “zones”, around the catalytic sites in these proteins [1,2,3,4]. Each zone incorporated a segment of the catalytic core, connected to their respective element of protein functional machinery through a network of conserved hydrogen bonds and other interactions.

The four protease superfamilies studied earlier were (1) alpha/beta-hydrolases, (2) trypsin-like serine proteases, (3) cysteine proteinases, and (4) SGNH hydrolase-like proteins (SCOP (Structural Classification of Proteins, https://scop.mrc-lmb.cam.ac.uk/; accessed on 1 March 2024 [5]) IDs: 3000102, 3000114, 3001808, and 3001315, respectively). Each had only rare, structural exceptions, where aspartic acid could be found in place of the canonical catalytic serine or cysteine residues. At the same time, most of the proteases that predominantly use aspartic acid as a catalytic residue are grouped into the “acid proteases” superfamily (SCOP ID: 3001059). This superfamily belongs to the “all beta proteins” class (SCOP ID: 1000001) and includes four families, including the “pepsin-like” family (SCOP ID: 4002301). The 3D structure of a protein from the pepsin-like family consists of two similar beta barrel domains (N- and C-terminal) with one catalytic aspartate residue in each domain [6,7,8]. Aspartic proteases of this family use an activated water molecule bound to two conserved aspartate residues for hydrolysis of their peptide substrates. Enzymes of the pepsin-like family are synthesized as inactive zymogens (proenzymes), and later they are subsequently activated by cleavage of the N-terminal propeptide, and separate upon activation [9]. The protease 3D structures of the other three families resemble that of one of the structural domains of the peptidase from the “pepsin-like” family, and they become active when two monomers assemble to form the catalytically active dimer [10].

Here, we propose a general model of the conserved structural catalytic core (SCC) of aspartate proteases. Based on the “key” features of this model, we present a comparative structural analysis of 3D structures of superfamily representative domains in their zymogenic, free, and ligand-bound forms found in the Protein Data Bank (PDB [11,12]). In addition, we show a comparative structural analysis of SCC models obtained after dimerization of two identical amino acid chains of proteases or duplication of corresponding amino acid fragments within the same chain. Certain elements of catalytic mechanism are discussed only to highlight the role of shown residues, but the complete protein functional analysis is not within the scope of this manuscript.

## 2. Characterization of the Structural Catalytic Core of the Members of the Superfamily of Acid Proteases

### 2.1. Creating the Dataset of the Acid Proteases Superfamily Fold Proteins

The SCOP classification database [5] and the Protein Data Bank (PDB, http://www.rcsb.org/; accessed on 1 March 2024 [11,12]) were used to identify and retrieve 33 representative structures of proteins from the acid protease superfamily (SCOP ID: 3001059). Detailed descriptions of the protein structural information contained within this set of PDB files are given below.

Structure visualization and structural analysis of interactions between amino acids in proteins (hydrogen bonds, hydrophobic, other types of weak interactions) were performed using Maestro (Schrödinger Release 2023-1: Schrödinger, LLC, New York, NY, USA, 2021; https://www.schrodinger.com/user-announcement/announcing-schrodinger-software-release-2023-4; accessed on 1 March 2024) and software [13] to determine interatomic contacts, i.e., of ligand–protein contacts (LPCs) and contacts of structural units (CSUs).

Pairwise superpositions of representative structures were conducted using the Dali server (http://ekhidna2.biocenter.helsinki.fi/dali/; accessed on 1 March 2024) [14]. Weak hydrogen bonds from C-H•O contacts were identified, based on the criteria described in [15]. The π-π stacking and similar contacts were analyzed using the Residue Interaction Network Generator (RING, https://ring.biocomputingup.it/submit; accessed on 1 March 2024) [16]. Dimers were built using the “Protein interfaces, surfaces and assemblies” service PISA at the European Bioinformatics Institute (http://www.ebi.ac.uk/pdbe/prot_int/pistart.html; accessed on 1 March 2024) [17]. Figures were drawn with MOLSCRIPT [18].

Currently, according to the SCOP, the acid protease superfamily consists of four families: (1) Lpg0085-like (SCOP ID: 4001811), (2) retroviral protease (retropepsin) (SCOP ID: 4002288), (3) pepsin-like (SCOP ID: 4002301), and (4) dimeric aspartyl proteases (SCOP ID: 4004443), with more than 146 representative domains [5].

Representative 3D structures of this superfamily are tabulated in Table 1. Of the four families, only the pepsin-like family contains 3D structures of the zymogenic form of aspartic proteases. In addition to the SCOP database, we used data from the Proteopedia and the Uniprot databases (http://proteopedia.org/wiki/index.php/Main_Page; accessed on 1 March 2024 [19,20] and https://www.uniprot.org/; accessed on 1 March 2024 [21], respectively). Ten proenzyme structures were identified, and they are indicated with a “p” in Table 1. Since each 3D structure of the pepsin-like proenzymes contained two similar domains, both domains were separately analyzed at their catalytic regions, and thus Table 1 contains two lines for each PDB ID of a proenzyme labeled as “a” and “b”. For four proteins out of ten, in addition to coordinates of the zymogenic form, there were also available coordinates for both the ligand-free and ligand-bound forms, labeled in Table 1 with letters “c/d” and “e/f”, respectively. For three out of ten proteins, in addition to the coordinates of the zymogenic form, there were coordinates of only the ligand-bound form (i.e., “a”, “b”, “e”, and “f” only; rows N: 4, 6, and 7). And for the remaining three proteins, there were coordinates available only for the zymogenic form (i.e., “a” and “b” only; rows N: 8–10). In addition to these ten proteases from the pepsin-like family, three proteolytically nonfunctional proteins in one or two forms were also analyzed (rows N: 11–13). The proteolytic inactivity of the last three proteins is caused by the replacement of their catalytic aspartic acids in the C-domains with serine.

In SCOP, the retroviral protease (retropepsin) family is represented by the 3D structures of proteases from ten different organisms: HIV-1, HIV-2, HTLV-1, M-PMV, FIV, XMRV, SIV, RSV, MAV, and EIAV [5]. Of the ten proteases listed, only the 3D structure of the XMRV protease differs from that of the other retropepsins [22,23]. Therefore, only the 3D structures of HIV-1 and XMRV proteases in the free and ligand-bound forms were chosen for analysis (Table 1, rows 14 and 15).

The dimeric aspartyl protease family contains seven representative protein 3D structures [5]. Six of the seven representative proteins are homologues of the DNA damage-inducible protein 1 (Ddi1) protease (PDB ID: 4Z2Z) [24]. The fold of the seventh representative protein, RC1339/APRc from *Rickettsia conorii* (PDB ID: 5C9F), does not form the mandatory homodimer like all other proteins in the dimeric aspartyl protease family [25]. Therefore, two 3D structures from this family, Ddi1 and APRc, were taken for conformational analysis. Finally, the Lpg0085-like family contains only one representative 3D structure (PDB ID: 2PMA) [26] and it was included in the analysis.

### 2.2. Structural Catalytic Core around the Catalytic Aspartates in Pepsin

Let us consider three variants of the pepsin 3D structure: the zymogenic propepsin (PDB ID: 3PSG), free pepsin (PDB ID: 4PEP), and ligand-bound pepsin (PDB ID: 6XCZ), which structurally define the pepsin-like family (SCOP ID: 4000470) (Table 1, rows 1a–1f).

**Table 1:** Structural amino acid alignment of the structural catalytic core (SCC) in the acid proteases superfamily proteins.

N	PDB ID and Chain	R(Å)	Protein	EC: Number	Propept. or N-Term Pept.	DD-Link	D-Loop	G-Loop	Mediator	Ref.
Superfamily: acid proteases										
Family: pepsin-like										
1a	3PSG_A,p	1.65	Propepsin	EC:3.4.23.1	7p VRK 9p	11 DTEY 14	31 F**D**TGSS 36	121 L**G**LA 124	Y125	[27]
1b	3PSG_A	1.65	Propepsin	-׀׀-		188 GYW 190	214 V**D**TGTS 219	301 L**G**DV 304		
1c	4PEP_A	1.80	Pepsin	-׀׀-	7 ENY 9	12 TEY 14	31 F**D**TGSS 36	121 L**G**LA 124	Y125	[28]
1d	4PEP_A	1.80	Pepsin	-׀׀-		188 GYW 190	214 V**D**TGTS 219	301 L**G**DV 304		
1e	6XCZ_A	1.89	Pepsin	-׀׀-	7 ENY 9	12 TEY 14	31 F**D**TGSS 36	121 L**G**LA 124	Y125	[29]
1f	6XCZ_A	1.89	Pepsin	-׀׀-		188 GYW 190	214 V**D**TGTS 219	301 L**G**DV 304		
2a	3VCM_A,p	2.93	Prorenin	EC:3.4.23.15	14p KRM 16p	11 DTQY 14	31 F**D**TGSS 36	121 V**G**MG 124	F125	[30]
2b	3VCM_A	2.93	Prorenin	-׀׀-		188 GVW 190	214 V**D**TGAS 219	301 L**G**AT 304		
2c	2REN_A	2.50	Renin	-׀׀-	13 TNY 15	18 TQY 20	37 F**D**TGSS 42	128 V**G**MG 131	F132	[31]
2d	2REN_A	2.50	Renin	-׀׀-		199 GVW 201	225 V**D**TGAS 230	315 L**G**AT 318		
2e	3K1W_A	1.50	Renin	-׀׀-	13 TNY 15	18 TQY 20	37 F**D**TGSS 42	128 V**G**MG 131	F132	[32]
2f	3K1W_A	1.50	Renin	-׀׀-		199 GVW 201	225 V**D**TGAS 230	315 L**G**AT 318		
3a	1PFZ_A,p	1.85	Proplasmepsin 2	EC:3.4.23.39	85p KVE 87p	12 QNIM 15	33 L**D**TGSA 38	124 L**G**LG 127	W128	[33]
3b	1PFZ_A	1.85	Proplasmepsin 2	-׀׀-		191 LYW 193	213 V**D**SGTS 218	301 L**G**DP 304		
3c	1LF4_A	1.90	Plasmepsin 2	-׀׀-	9 VDF 11	14 IMF 16	33 L**D**TGSA 38	124 L**G**LG 127	W128	[34]
3d	1LF4_A	1.90	Plasmepsin 2	-׀׀-		191 LYW 193	213 V**D**SGTS 218	301 L**G**DP 304		
3e	2BJU_A	1.56	Plasmepsin 2	-׀׀-	9 VDF 11	14 IMF 16	33 L**D**TGSA 38	124 L**G**LG 127	W128	[35]
3f	2BJU_A	1.56	Plasmepsin 2	-׀׀-		191 LYW 193	213 V**D**SGTS 218	301 L**G**DP 304		
4a	3QVC_A,p	2.10	HAP zymogen	EC:3.4.23.39	84p NIE 86p	9 LANVL 13	31 FHTASS 36	121 F**G**LG 124	W125	[36]
4b	3QVC_A	2.10	HAP zymogen	-׀׀-		188 LMW 190	214 L**D**SATS 219	301 L**G**DP 304		
4e	3QVI_A,B	2.50	HAP protein	-׀׀-	7_B K	12 VLS 14	31 FHTASS 36	121 F**G**LG 124	W125	[36]
4f	3QVI_A	2.50	HAP protein	-׀׀-		188 LMW 190	214 L**D**SATS 219	301 L**G**DP 304		
5a	5N7N_A,p	2.30	Procathepsin D	N/A	7p TRF 9p	37 DVVY 40	57 F**D**TGSA 62	147 L**G**LA 150	Y151	[37]
5b	5N7N_A	2.30	Procathepsin D	-׀׀-		217 GYW 219	248 ANTGTS 253	336 L**G**DV 339		
5c	5N71_A	1.88	Cathepsin D	-׀׀-	33 VNL 35	38 VVY 40	57 F**D**TGSA 62	147 L**G**LA 150	Y151	[37]
5d	5N71_A	1.88	Cathepsin D	-׀׀-		217 GYW 219	248 ANTGTS 253	336 L**G**DV 339		
5e	5N7Q_A	1.45	Cathepsin D	-׀׀-	11 VNL 13	16 VVY 18	35 F**D**TGSA 40	125 L**G**LA 128	Y129	[37]
5f	5N7Q_A	1.45	Cathepsin D	-׀׀-		195 GYW 197	226 A**D**TGTS 231	314 L**G**DV 317		
6a	1MIQ_A,p	2.50	Proplasmepsin	N/A	84p KVE 86p	13 NIM 15	33 F**D**TGSA 38	124 L**G**LG 127	W128	[38]
6b	1MIQ_A	2.50	Proplasmepsin	-׀׀-		191 LYW 193	213 V**D**SGTT 218	301 L**G**DP 304		
6e	1QS8_A	2.50	Plasmepsin	-׀׀-	9 DDV 11	14 IMF 16	33 F**D**TGSA 38	124 L**G**LG 127	W128	[38]
6f	1QS8_A	2.50	Plasmepsin	-׀׀-		191 LYW 193	213 V**D**SGTT 218	301 L**G**DP 304		
7a	5JOD_A,p	1.53	Proplasmepsin 4	EC:3.4.23.39	85p KID 87p	13 NLM 15	33 F**D**TGSA 38	124 L**G**LG 127	W128	[39]
7b	5JOD_A	1.53	Proplasmepsin 4	-׀׀-		191 LYW 193	213 V**D**SGTS 218	301 L**G**DP 304		
7e	1LS5_A	2.80	Plasmepsin 4	-׀׀-	9 DDV 11	14 LMF 16	33 F**D**TGSA 38	124 L**G**LG 127	W128	[34]
7f	1LS5_A	2.80	Plasmepsin 4	-׀׀-		191 LYW 193	213 V**D**SGTS 218	301 L**G**DP 304		
8a	1QDM_A,p	2.30	Prophytepsin	EC:3.4.23.40	11p KKR 13p	15 NAQY 18	35 F**D**TGSS 40	126 L**G**LG 129	F130	[40]
8b	1QDM_A	2.30	Prophytepsin	-׀׀-		195 GYW 197	222 A**D**SGTS 227	313 L**G**DV 316		
9a	1HTR_B,p	1.62	Progastricsin	EC:3.4.23.3	8p KKF 10p	11 DAAY 14	31 F**D**TGSS 36	121 M**G**LA 124	Y125	[41]
9b	1HTR_B	1.62	Progastricsin	-׀׀-		189 LYW 191	216 V**D**TGTS 221	304 L**G**DV 307		
10a	1TZS_A,p	2.35	Procathepsin E	EC:3.4.23.34	9p R	22 DMEY 25	42 F**D**TGSS 47	132 L**G**LG 135	Y136	[42]
10b	1TZS_A	2.35	Procathepsin E	-׀׀-		201 AYW 203	227 V**D**TGTS 232	317 L**G**DV 320		
11c	1T6E_X	1.70	Xylanase inhib.	EC:3.2.1.8	8 TKD 10	14 SLY 16	28 L**D**VAGP 33	141 A**G**LA 144	NS146	[43]
11d	1T6E_X	1.70	Xylanase inhib.	-׀׀-		204 PAH 206	234 LSTRLP 239	348 L**G**GA 351		
11e	1T6G_A	1.80	Xylanase inhib.	-׀׀-	8 TKD 10	14 SLY 16	28 L**D**VAGP 33	141 A**G**LA 144	NS146	[43]
11f	1T6G_A	1.80	Xylanase inhib.	-׀׀-		204 PAH 206	234 LSTRLP 239	348 L**G**GA 351		
12c	3AUP_A	1.91	Basic 7S globulin	N/A	15 QND 17	21 GLH 23	40 V**D**LNGN 45	159 A**G**LG 162	HA164	[44]
12d	3AUP_A	1.91	Basic 7S globulin	-׀׀-		228 GEY 230	264 ISTSTP 269	361 L**G**AR 364		
13c	3VLA_A	0.95	EDGP (Fragment)	N/A	14 KKD 16	20 LQY 22	39 V**D**LGGR 44	155 A**G**LG 158	RT160	[45]
13d	3VLA_A	0.95	EDGP (Fragment)	-׀׀-		235 VEY 237	270 ISTINP 275	374 I**G**GH 377		
13e	3VLB_A	2.70	EDGP (Fragment)	-׀׀-	14 KKD 16	20 LQY 22	39 V**D**LGGR 44	155 A**G**LG 158	RT160	[46]
13f	3VLB_A	2.70	EDGP (Fragment)	-׀׀-		235 VEY 237	270 ISTINP 275	374 I**G**GH 377		
Family: retroviral protease (retropepsin)										
14c	3IXO_A	1.70	HIV-1 protease	N/A	N/A	8 R-P 9	24 L**D**TGAD 29	85 I**G**RN 88	N/A	[46]
14d	3IXO_B	1.70	HIV-1 protease	-׀׀-	N/A	8 R-P 9	24 L**D**TGAD 29	85 I**G**RN 88	N/A	
14e	5YOK_A	0.85	HIV-1 protease	-׀׀-	N/A	8 R-P 9	24 L**D**TGAD 29	85 I**G**RN 88	N/A	[47]
14f	5YOK_B	0.85	HIV-1 protease	-׀׀-	N/A	8 R-P 9	24 L**D**TGAD 29	85 I**G**RN 88	N/A	
15c	3NR6_A	1.97	XMRV protease	EC:3.4.23.-	N/A	15 E-P 16	31 V**D**TGAQ 36	93 L**G**RD 96	R95	[22]
15d	3NR6_B	1.97	XMRV protease	-׀׀-	N/A	15 E-P 16	31 V**D**TGAQ 36	93 L**G**RD 96	R95	
15e	3SLZ_A	1.40	XMRV protease	N/A	N/A	15 E-P 16	31 V**D**TGAQ 36	93 L**G**RD 96	R95	[48]
15f	3SLZ_B	1.40	XMRV protease	-׀׀-	N/A	15 E-P 16	31 V**D**TGAQ 36	93 L**G**RD 96	R95	
Family: dimeric aspartyl proteases										
16c	4Z2Z_A	1.80	Ddi1 protease	EC:3.4.23.-	N/A	201 VPML 204	219 V**D**TGAQ 224	289 I**G**LD 292	N/A	[49]
16d	4Z2Z_B	1.80	Ddi1 protease	-׀׀-	N/A	201 VPML 204	219 V**D**TGAQ 224	289 I**G**LD 292	N/A	
17c	5C9F_A	2.00	ApRick protease	EC:3.-.-.-	N/A	121 DGHF 124	139 V**D**TGAS 144	209 L**G**MS 212	N/A	[25]
Family: LPG0085-like										
18c	2PMA_A	1.89	Protein Lpg0085	N/A	N/A	29 Y	46 L**D**TGAK 51	145 L**G**RD 148	RD148	[26]
18d	2PMA_I	1.89	Protein Lpg0085	-׀׀-	N/A	29 Y	46 L**D**TGAK 51	145 L**G**RD 148	RD148	

N/A—Not Available.

The boundary between the N- and C-domains of the 3D structure of pepsinogen is in the vicinity of Gly_169_ [9]. Asp_32_ (N-domain) and Asp_215_ (C-domain) are the two catalytically important aspartate residues. Each aspartate residue is positioned within the hallmark Asp-Thr/Ser-Gly (Asp_32_-Thr_33_-Gly_34_ in 3PSG) motif which, together with a further Hydrophobic-Hydrophobic-Gly sequence motif, forms an essential structural feature known as a psi-loop motif [28,50,51,52,53]. Let us designate two fragments of the protease amino acid sequence involved in formation of the psi-loop motif as the D(Asp)-loop and G(Gly)-loop. In this section, the atomic structure of the D- and G-loops in the N- and C-domains and their position relative to each other in the 3D structures of pepsin will be analyzed in detail.

#### 2.2.1. Propepsin

##### DD-Zone of Propepsin: A D-Loop_N_ - DD-Link_N_ - D-Loop_C_ - DD-Link_C_ Circular Motif

**Figure 1:**
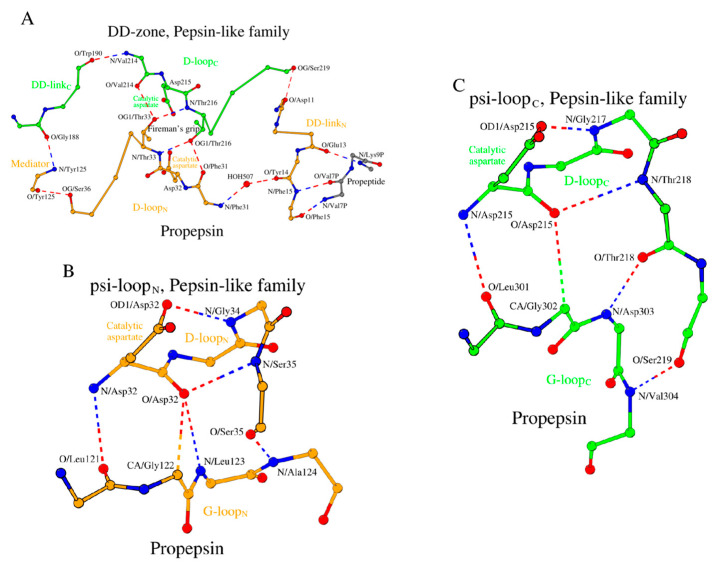
Three building blocks of the structural catalytic core (SCC) in propepsin (PDB ID: 3PSG), as a representative member of the pepsin-like family of the acid protease superfamily. (**A**) DD-zone, (**B**) psi-loop_N_, and (**C**) psi-loop_C_. The dashed lines show long-range hydrogen bonds between the bordering amino acids of fragments of the primary structure of the protein: D-loops, DD-link, mediator, and G-loops, thus determining the cyclic nature and composition of the residues of each block separately. A dimer of dipeptides, Asp_32_-Thr_33_ and Asp_215_-Thr_216_, from two D-loops, form the fireman’s grip in the DD-zone, which is characterized by four long-range hydrogen bonds, while tetrapeptides, Asp_32_-...-Ser_35_ and Asp_215_-...-Thr_218,_ from two D-loops, form the Asx-motif in psi-loop_N_ and psi-loop_C_, which is characterized by two short-range hydrogen bonds. Structural differences in two long-range hydrogen bonds located within psi-loop_N_ (O/Asp_32_-N/Leu_123_ and (O/Ser_35_-N/Ala_124_) and psi-loop_C_ (O/Thr_218_-N/Asp_303_ and O/Ser_219_-N/Val_304_) influence the functional differences between the catalytic aspartates.

As noted above, the functional activity of pepsin is carried out simultaneously by both of the catalytic residues, Asp_32_ and Asp_215_. Therefore, two D-loops, D-loop_N_ for the N-terminal domain and D-loop_C_ for the C-terminal domain, were analyzed in detail (Table 1 and Table S1). It was found that the two domains of propepsin also contain structurally equivalent short peptides, which we call DD-link_N_ (Asp_11_-...-Tyr_14_) and DD-link_C_ (Gly_188_-Tyr_189_-Trp_190_), where N and C also stand for the N-terminal domain and C-terminal domain, respectively (Table 1). These two special DD-link peptides “lock” the ends of the D-loop_N_ and D-loop_C_ to form a “circular” structure, which altogether we call the “DD-zone” (Figure 1A).

The DD-zone of propepsin consists of 19 amino acids in total from both D-loops and both DD-links and an additional residue Tyr_125_. Tyr_125_ serves as a structural mediator between the C-terminus of the D-loop_N_ and the N-terminus of the DD-link_C_ (Figure 1A); this residue directly follows Ala_124_ from G-loop_N_ (Table 1).

Independently, in propepsin, residues Thr_33_ and Thr_216_ are located next to the two catalytic aspartates. Their side-chain OG1 atoms each make two hydrogen bonds with main-chain nitrogen and oxygen atoms of the opposite D-loop (Figure 1A, Table S1, last column). These interactions are known as the “fireman’s grip” motif [54,55].

The proenzyme segment in propepsin is Leu_1p_-...-Leu_44p_, where “p” indicates the proenzyme sequence region. The pepsin portion in 3PSG starts from Ile_1_. Glu_13_ and Phe_15_ form a short β-sheet-like interaction with Lys_9p_ and Val_7p_ (Figure 1A, Table S2, last column). The residues of this β-sheet undergo a conformational change during the activation process [9].

##### The Psi-Loop_N_ and Psi-Loop_C_ Motifs: Interactions between the D-Loop and G-Loop in the N- and C-Domains

In 3PSG, the D-loop_N_ tetrapeptide, Asp_32_ -...- Ser_35,_ contains a frequently occurring Asx-motif [56], where an aspartate (here, catalytic Asp_32_) or an asparagine residue within a tetra- or pentapeptide forms two short-range (in terms of sequence location) main-chain and side-chain hydrogen bonds with the sequentially adjacent amino acids (Figure 1B). We observe a similar Asx-motif involving the catalytic Asp_215_ from the D-loop_C_ tetrapeptide (Figure 1C). Additionally, there are four conserved long-range hydrogen bonds between the D- and G-loops in both N- and C-domains (Figure 1B,C). We will refer to the substructures shown in Figure 1B,C as the psi-loop_N_ and psi-loop_C_ motifs. Each psi-loop motif is an eight-residue 3D structure consisting of D- and G-loop residues that are held together by six hydrogen bonds. The geometric characteristics of these six hydrogen bonds are given in Table S2 (row 1a, columns 4–6).

##### Comparison of the Psi-Loop_N_ and Psi-Loop_C_

Despite the apparent similarity, the psi-loop_N_ and psi-loop_C_ motifs are not identical. While making similar interactions, the D-loop_C_ is five amino acids long (Asp_215_-...-Ser_219_) and the D-loop_N_ has only four residues (Figure 1B,C). Moreover, the conformations of the two respective G-loops differ. The G-loop_C_ at its C-terminus contains a β-turn, which is stabilized by the hydrogen bond between O/Gly_302_ and N/Phe_305_, while the G-loop_N_ does not have a similar substructure. As a result, there is conformational difference between Phe_305_ and its structural counterpart in the N-domain, Tyr_125_, where Phe_305_ takes part in the conformational arrangement of its respective psi-loop, while Tyr_125_ does not. Still, the two psi-loop motifs are bound by a set of equivalent interactions, where the O/Asp_32_-N/Leu_123_ hydrogen bond in psi-loop_N_ is substituted by the O/Thr_218_-N/Asp_303_ hydrogen bond in psi-loop_C_, and where the O/Ser_35_-N/Ala_124_ hydrogen bond in psi-loop_N_ is substituted by the O/Ser_219_-N/Val_304_ hydrogen bond in psi-loop_C_ (Figure 1B,C).

The structural changes described above appear to result in tighter binding of Asp_32_ to the G-loop_N_ than of Asp_215_ to G-loop_C_, since the distance from Asp_32_ to G-loop_N_ is shorter than that from Asp_215_ to G-loop_C_. It is possible that this structural fact is the main reason for the differences in functional activity between Asp_32_ and Asp_215_ in the proposed models of catalytic hydrolysis of peptide bonds by acid proteases [57,58,59]. If Asp_32_ is more tightly bound with more potential hydrogen bonds as compared to Asp_215_, then its nucleophilicity must be somewhat decreased. Thus, Asp_215_ of the C-domain would play a more prominent role in the proteolytic cleavage of dipeptide substrates than Asp_32_ of the N-domain.

**Figure 2:**
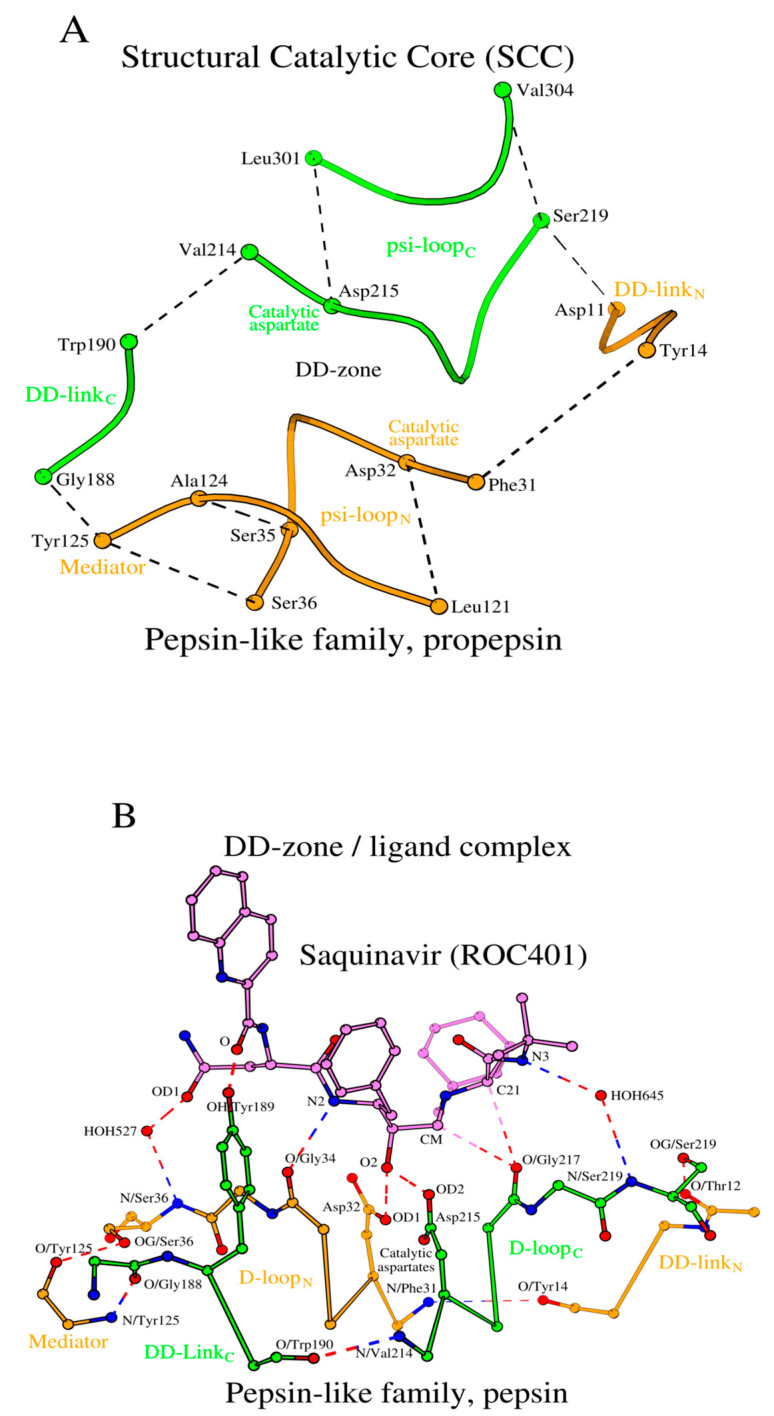
Interface organization of interactions between the SCC of pepsin and the ligand saquinavir. (**A**) A smooth coil representation is shown that passes through the CA atom positions of the pepsin’s SCC. The dashed lines show the complete set of long-range hydrogen bonds between the bordering residues of the six amino-acid sequence fragments. (**B**) The potential hydrogen bonding interactions between the D-loops of the DD-zone and saquinavir are shown with dashed lines.

The structural association of two psi-loops and the DD-zone allows us to obtain an assembly of structural elements of the structural catalytic core (SCC) of propepsin (Figure 2A). It includes all 28 amino acids listed in Table 1 (rows 1a and 1b).

#### 2.2.2. Activation of Free Pepsin

The conversion of propepsin to active pepsin is achieved through proteolytic cleavage and subsequent removal of the N-terminal amino acid fragment. Here, we are mostly interested in changes that occur in the propepsin structural core, SCC. A structural comparison of propepsin (PDB ID: 3PSG) and mature pepsin (PDB ID: 4PEP) showed that rearrangements occur only in DD-link_N_ and its immediate environment. First, as described above, the length of the tetrapeptide Asp_11_-...-Tyr_14_ was reduced by one residue at its N-terminus (Table 1 and Table S1). Then, the two-stranded β-sheet (Glu_13_-...-Phe_15_)/(Val_7p_-...-Lys_9p_) is replaced with a structurally similar two-stranded β-sheet (Glu_13_-...-Phe_15_)/(Glu_7_-...-Tyr_9_) (Table 1 and Table S2). Thus, upon pepsin activation the architecture of the SCC remains largely unchanged.

#### 2.2.3. Pepsin/Ligand Complex

During activation, the propepsin structure transforms into the active pepsin structure, ligand-free form. How does interaction with the ligand affect the SCC? Let us consider the 3D structure of the pepsin/saquinavir complex (PDB ID: 6XCZ). The key contacts between pepsin and the small-molecule ligand (saquinavir, ROC_401_) are four hydrogen bonds (Figure 2B; Table S3, rows 1e and 1f). Two pairs of conserved residues from the D-loops of the N- and C-domains, Asp_32_/Gly_34_ and Asp_215_/Gly_217_, donate four oxygen atoms as part of the four hydrogen bonds. Each of the two aspartates forms an Asx-motif [56], and in addition to the four hydrogen bonds above, there are two additional hydrogen bonds via the mediator-waters HOH_527_ and HOH_645_ (Figure 2B), and also there is a hydrogen bond that involves the OH atom of Tyr_189_, the central residue of the tripeptide DD-link_C_. Thus, DD-link_C_ interacts with the inhibitor. Aside from the extensive hydrogen bonding inventory described above, binding of a ligand does not introduce any visible structural changes to the ligand-free form of the SCC of pepsin (Tables S1 and S2, rows 1c–1f).

**Figure 3:**
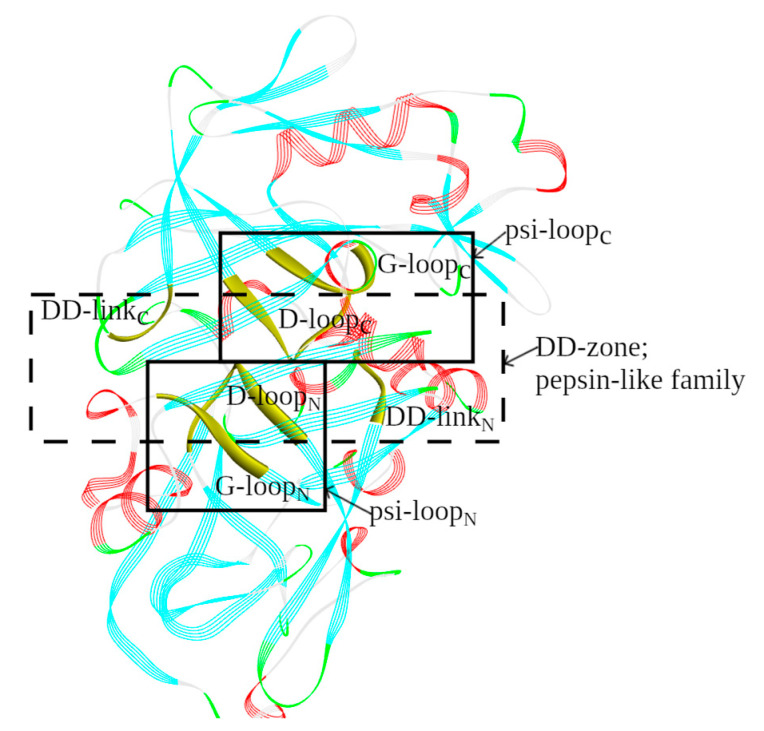
The 3D structure of the active site in pepsin-like family aspartic proteases. The three boxes show the location of the structural catalytic core (SCC) in propepsin (PDB ID: 3PSG_A). It consists of a DD-zone (a central rectangle constructed using dotted lines) and two psi-loops (solid lines). The discussed structural elements (loops and links) are highlighted and labeled.

The location of the structural catalytic core (SCC) in the 3D structure of propepsin is shown in Figure 3.

### 2.3. Structural Core in Proteins of the Pepsin-like Family

#### 2.3.1. DD-Zones

Earlier, we showed that in propepsin the segment Asp_11_-Phe_15_, which includes DD-link_N_, interacts with the pro-tripeptide Val_7p_-Lys_9p_ (Figure 1A) by means of interactions listed in Table S2. During the transition from the inactive zymogenic form to the enzymatically active form, DD-link_N_ is slightly structurally modified as described above, and the pro-tripeptide is spatially substituted by the N-terminal tripeptide (Glu_7_-Tyr_9_; Table 1). Interactions between DD-link_N_ and the N-terminal tripeptide are shown in Table S2. We also observed similar structural rearrangements in the other members of the pepsin-like family although there are variations from the rule: with the histo-aspartic protease (HAP), DD-link_N_ is one amino acid longer, and with procathepsin E, only one amino acid, R_9P_, of the propeptide, contacts DD-link_N_ (Table 1). However, the general structural trend for the pepsin-like family is the same.

In propepsin and pepsin, the contact between DD-link_N_ and D-loop_N_ involves a water molecule as an intermediary (Figure 1; Table S1). In the structure of ligand-bound pepsin, a water molecule does not participate in interactions as an intermediary. A similar water presence and functionality is observed for all of the remaining proteins of the pepsin-like family. However, considering differences in the resolution of structures (Table 1) and the associated difficulties in localization of the bound water molecules, it is not always possible to unambiguously correlate the presence or absence of a water molecule with any form of protein, and thus exceptions are possible.

In pepsin, the contact between D-loop_N_ and DD-link_C_ involves the amino acid Tyr_125_ as a structural mediator (Figure 1; Table S1). In a number of proteins, there is also a mediating water molecule in addition to the aromatic amino acid (Table S1, column 5). In three proteins, xylanase inhibitor, basic 7S globulin, and EDGP, there are two mediator residues instead of a single Tyr_125_. A hydrogen bond between the ends of DD-link_C_ and D-loop_C_ is, however, conserved and contains no mediator insertions in any of the analyzed structures (Table S1, column 6). The contact between D-loop_C_ and DD-link_N_ does not contain mediators, but can be variable in its nature, being a hydrogen bond, a weak hydrogen bond, or a hydrophobic interaction (Table S1, column 7).

##### Fireman’s Grip Motif Reflects Open/Close-Conformation Structural Change

In the pepsin-like family proteins, the open/close-conformation structural change during the transition from the inactive zymogen to the enzymatically active form can either lead to conformational changes in the DD-zone or not. In proteins, where the hallmark Asp-Thr/Ser-Gly sequence (see Section 2.2) in the C-terminal domain contains serine, the conformational change in the DD-zone does take place, and it is reflected by the change of the fireman’s grip motif (Table S1, column 8). In proteins, where the hallmark Asp-Thr/Ser-Gly sequence in the C-terminal domain contains threonine, the open/close conformational change in the DD-zone does not take place.

#### 2.3.2. Psi-Loops

As noted above, the psi-loop motif includes amino acids from the D- and G-loops. In pepsin, both D-loops contain a catalytic aspartate. Of the thirteen proteins studied, eight are active hydrolases, and have both catalytic aspartates (Table 1). In the HAP protein, an evolutionary Asp_32_His mutation did occur, which, however, did not lead to a loss of catalytic activity because the other Asp_215_ was still present [36]. The remaining four proteins, cathepsin D, xylanase inhibitor, basic 7S globulin, and EDGP, lost their enzymatic activity due to the replacement of the catalytic aspartate with another amino acid in the C-terminal domain [37,43,44,45]. Loss of catalytic activity in these proteins versus the HAP protein is strong evidence that proteolytic activity requires the aspartate of the C-terminal domain, whereas the aspartate of the N-terminal domain may be dispensable.

Both psi-loop_N_ and psi-loop_C_ motifs are structurally identical among the thirteen proteins of the pepsin-like family in three different forms (proenzyme, mature enzyme, and enzyme/ligand complex) (Table S2, columns 4 and 5). That is, replacing the catalytic aspartate with another amino acid either does not affect the conformation of the psi-loop motifs or affects it insignificantly. Structural conservation of the psi-loop conformation also occurs despite structural rearrangement in the tetrapeptides forming the Asx-motif in some proteins (Table S2, column 6). For example, six proteins in one or several forms show a structural transition from the Asx-motif to a Asx-turn [60], which lacks the hydrogen bond between the atoms of the first and fourth residues of the tetrapeptide unlike the Asx-motif. The structures of these six proteins, the HAP protein, plasmepsin 4, phytepsin, xylanase inhibitor, basic 7S globulin, and EDGP, have geometrical parameters that formally exceed those of a canonical hydrogen bond [61].

#### 2.3.3. Ligand Bound Pepsin-like Proteins

Section 2.2.3 identifies seven amino acids of the pepsin’s SCC that are responsible for ligand recognition. These are (1, 2, 3 and 4) catalytic Asp/Gly pairs of (Asp-Thr/Ser-Gly)_N_ and (Asp-Thr/Ser-Gly)_C_, C-terminal and N-terminal Asp-Thr/Ser-Gly motifs; (5 and 6) two C-terminal serine residues of D-loop_N_ and D-loop_C_; and (7) the Tyr_189_, the central residue of the tripeptide DD-link_C_. Of the thirteen pepsin-like representative structures listed in Table 1, only seven had a complex with a ligand close to or within the SCC. Six of these seven structures had similar D-loop/ligand contacts (Table S3). And, again, the HAP protein was unique, by lacking the expected contacts of Ala_217_ and Ser_219_ with the K95 inhibitor as seen in all of the other structures. With the HAP protein, instead of those contacts, Ala_217_ and Ser_219_ of chain_A formed hydrogen bonds with Asn_279_ of chain_B, i.e., O/Ala_217_A_ - N/Asn_279_B_ at 2.9 Å and OG/S_219_-ND2/N_279_B_ at 3.1 Å, respectively, and a weak hydrogen bond with Glu_278A_ of chain_B (designated as Glu_278A_B_ in the PDB file of 3QVI), O/Ala_217_A_ - CA/Glu_278A_B_ at 3.4 (2.6) 127° (for the definition of parameters of weak hydrogen bonds, see [15]). The changes in contact partners for Ala_217_ and Ser_219_ are due to the fact that in the inhibitor complex the enzyme forms a tight domain-swapped dimer, not previously seen in any aspartic protease [36]. As a result of such domain-swapped dimerization, Glu_278A_ of chain_B forms contacts with the inhibitor instead of Ala_217_ and Ser_219_ of chain_A (Table S3, row 4f and column 5).

Taken together, the pepsin-like family proteins from Table 1 have their SCC constructed from the same set of conserved amino acids in all three forms, i.e., proenzyme, ligand-free enzyme, and ligand-bound enzyme, while the most noticeable structural changes concern the transition of the DD-links and fireman’s grips from the zymogenic form to the enzymatic form. The DD-zones include the N-terminal and C-terminal D-loops, D-loop_N_ and D-loop_C_, with their ends linked by the longer DD-link_N_ and a water molecule, and a shorter DD-link_C_ plus a mediator molecule (Figure 1A).

### 2.4. SCC in Hydrolases of the Retroviral Protease (Retropepsin) Family

#### 2.4.1. DD-Zones

**Figure 4:**
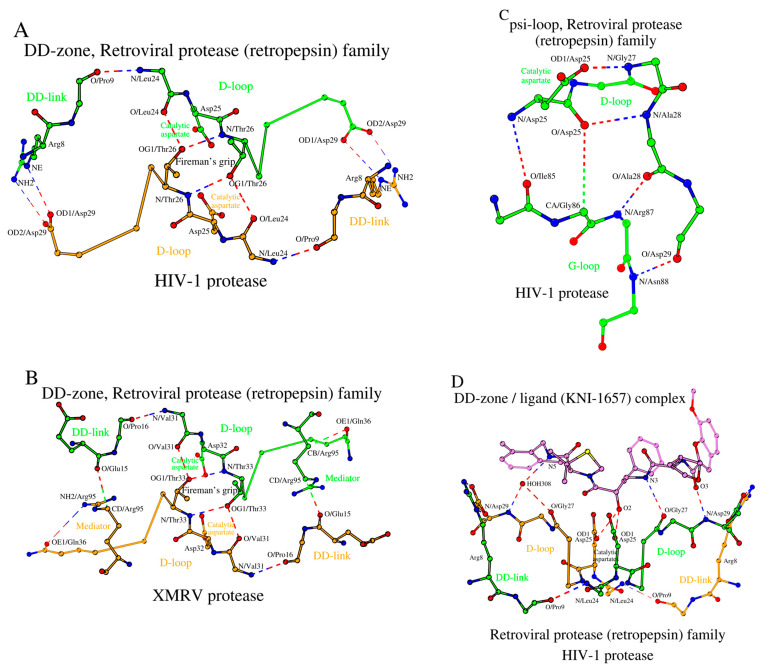
The building blocks of the SCC in the HIV-1 and XMRV homodimer proteases (PDB IDs: 3IXO and 3NR6, correspondingly), as the representative members of the retroviral protease (retropepsin) family of the acid protease superfamily. (**A**) DD-zone of HIV-1 protease, (**B**) DD-zone of XMRV protease, and (**C**) psi-loop of HIV-1 protease. (**D**) The potential hydrogen bonding interactions (dashed lines) between two identical D-loops of the DD-zone and the ligand in the HIV-1 protease with inhibitor KNI-1657 complex (PDB ID: 5YOK).

The retroviral protease (retropepsin) family is the second family of acid proteases listed in Table 1. Hydrolases of this family do not have a zymogenic form, and the enzyme is a dimer of two identical amino acid chains. Figure 4A shows a DD-zone of HIV-1 protease (PDB ID: 3IXO). The main differences between the DD-zones of pepsin and HIV-1 are the number of residues forming DD-links and an absence of mediators.

A change in the number of residues in the DD-links is usually associated with the presence or absence of the need to form a β-structural contact with either the propeptide or the N-terminal fragment (Figure 4A vs. Figure 1A). However, a decrease in the length of the DD-link by one amino acid does not necessarily lead to a change in the relative position of the D-loops relative to each other. Such is the case for the HIV-1 protease, where atoms of the long side-chain of Arg_8_ (DD-link in HIV-1) interact with Asp_29_ (D-loop in HIV-1) instead of the oxygen atoms of the shorter side-chains of Asp_11_ (DD-link in pepsin) and Ser_219_ (D-loop in pepsin) (Figure 4A vs. Figure 1A, Table S1).

In the XMRV protease (PDB ID: 3NR6), there is glutamate (DD-link in XMRV) in place of Arg_8_ (DD-link in HIV-1) and glutamine (D-loop in XMRV) instead of Asp_29_ (D-loop in HIV-1) (Table 1), which results in some changes in the architecture of the DD-zone in the XMRV protease compared to HIV-1 (Figure 4B, Table S1). In XMRV, there is an increase in the distance between the ends of the DD-link and the D-loop, which results in the absence of a direct contact between them. However, in XMRV, the D-loop/DD-link contact happens through the mediator residue Arg_95_, which also participates in the formation of the psi-loop (Figure 4B).

Thus, the distinctive feature of the retroviral protease (retropepsin) family hydrolases is within the DD-zones, where the D-loops are bound by short DD-links of two residues plus a mediator residue. Additionally, in HIV-1 and XMRV, there is a separate residue Arg_87_ (in HIV-1)/Arg_95_ (in XMRV), which interacts with Asp_29_ (in HIV-1)/Gln_36_ (in XMRV) via a conventional hydrogen bond: NH2/R_87_-OD1/D_29_ (Table S1, column 5), and stabilizes the conformation of the D-loop. The function of this residue in HIV-1 and XMRV is unknown.

#### 2.4.2. Psi-Loops in HIV-1 and XMRV

As noted above, a homodimer of two identical amino acid chains is the active form of a HIV-1 protease. Therefore, one can expect the conformation of the psi-loop motif in chains A and B to be identical. It was found out that HIV-1 and XMRV not only have similar psi-loop motifs, but they are also similar to that observed in the C-domain of pepsin (Figure 1C and Figure 4C). That is, the identical psi-loops in HIV-1 and XMRV have chosen a conformation that provides a catalytic aspartate with higher proteolytic efficiency in both subunits (Table S2). In Table S2, homodimer chains A and B in HIV-1 (and other retroproteases) are listed as the respective counterparts of the N- and C-domains in pepsin, but this is an arbitrary assignment.

#### 2.4.3. Ligand-Bound Forms of Retroviral Proteases

The DD-zones of ligand-bound pepsin and HIV-1 are very similar to each other (Figure 2B and Figure 4D). The main interactions are made by the three amino acids from each of the two D-loops, totaling six interacting residues (Table S3). In HIV-1, these residues are Asp_25_, Gly_27_, and Asp_29_ from D-loop of chain A and, of course, identical residues are in D-loop of chain_B of the HIV-1 homodimer (Figure 4D). For comparison, in pepsin, those amino acids are Asp_32_, Gly_34_, and Ser_36_ from D-loop_N_ and Asp_215_, Gly_217_, and Ser_219_ from D-loop_C_ (Table S3). In addition, with pepsin, Section 2.2.3 describes the additional Tyr_189_ from the DD-link_C_ that is involved in contacts with the ligand. In the ligand-bound HIV-1 protease (PDB ID: 5YOK), a combination of Arg_8_ (DD-link)/Asp_29_ (D-loop) performs an analogous role. Similar to HIV-1, in the ligand-bound XMRV (PDB ID: 3SLZ), the C-terminal position of the D-loop, Gln_36_, also participates in ligand binding (Table S3, last column). Replacing Asp_29_ (in HIV-1) with Gln_36_ (in XMRV) also results in additional hydrogen bonds formed between XMRV and the inhibitor. Interaction with the ligand does not seem to affect the architecture of the DD-zone in the HIV-1 and XMRV proteases (Table S1).

The X-ray structure of the retroviral HIV-1 protease (Figure 4D) shows an identical mode of interaction between two catalytic aspartates, Asp_25_ of chain_A and _B, and the bound ligand. However, if we take into account additional neutron crystallography data, we find that the catalytic aspartates are not identical in terms of their protonation state [62,63]. According to these data, one aspartate is protonated and the other is deprotonated at physiological pH. As a result, the two catalytic aspartates do interact differently with the same ligand. The deprotonated aspartate uses one of its deprotonated side-chain oxygens to interact with the hydrogen bound to the O2 atom of the ligand. At the same time, the protonated aspartate uses its protonated side-chain oxygen to interact directly with the same O2 oxygen atom of the ligand. These additional experimental data show the different roles that these two aspartates play in the catalytic mechanism of the HIV-1 protease.

**Figure 5:**
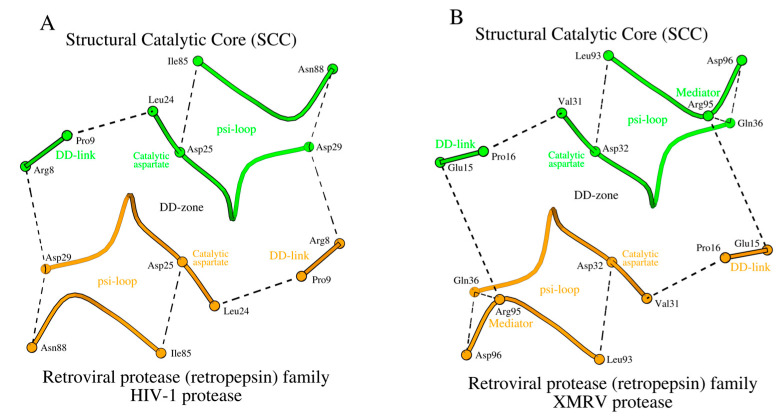
SCC of (**A**) HIV-1 and (**B**) XMRV proteases. A smooth coil representation is used in the figures, which passes through the CA atom of SCC positions of the corresponding retroviral proteases. The SCC of the XMRV protease differs from the SCC of the HIV-1 protease by the inclusion of the mediator residue Arg_95_ from the G-loop in each monomer.

The SCCs of the HIV-1 and XMRV proteases are shown in Figure 5A,B.

**Figure 6:**
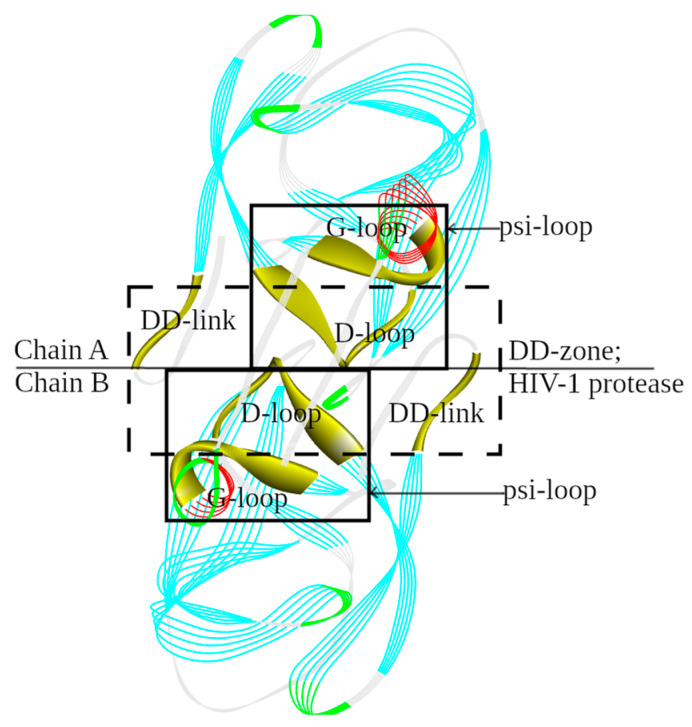
The 3D structure of the active site in retroviral protease (retropepsin) family aspartic proteases. The three boxes show the location of the structural catalytic core (SCC) in HIV-1 protease (PDB ID: 3IXO_A, B). It consists of a DD-zone (a central rectangle constructed using dotted lines) and two psi-loops (solid lines). The discussed structural elements (loops and links) are highlighted and labeled.

The location of the structural catalytic core (SCC) in the 3D structure of HIV-1 protease is shown in Figure 6.

### 2.5. SCCs of the Dimeric Aspartyl Proteases and Lpg0085-like Family Proteins

**Figure 7:**
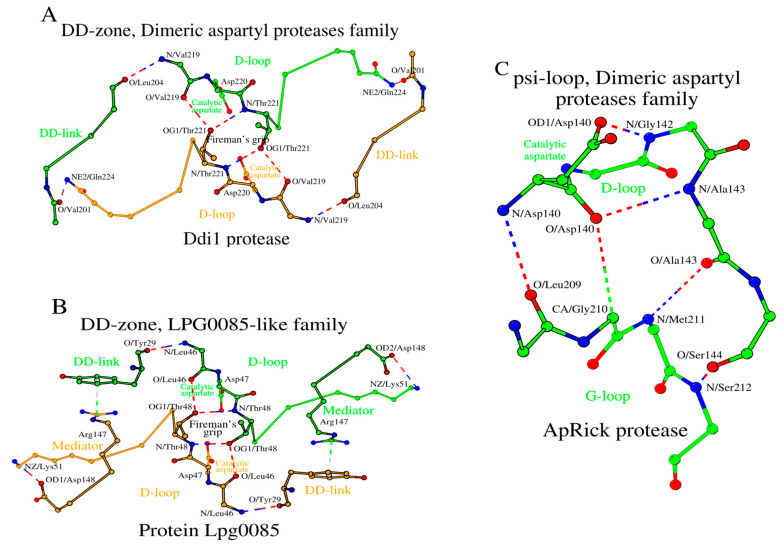
The building blocks of the SCC in the Ddi1 protease, Lpg0085 protein, and ApRick protease (PDB IDs: 4Z2Z, 2PMA and 5C9F, correspondingly), as the representative members of the dimeric aspartyl protease and LPG0085-like families of the acid protease superfamily. (**A**) DD-zone of Ddi1 protease, (**B**) DD-zone of protein Lpg0085, and (**C**) psi-loop of ApRick protease.

In HIV-1 and XMRV, we have shown how amino acid changes at the N-terminus of the DD-link and the C-terminus of the D-loop affect the structure of the DD-zone. The Ddi1 protease, like the XMRV protease, has glutamine as the C-terminal amino acid of the D-loop (Table 1 and Table S1, rows 16c and 16d). However, the DD-links of the Ddi1 and XMRV proteases differ in length. In Ddi1, the number of amino acids in the DD-link increases twofold (from 2 to 4 residues) compared to XMRV protease, while in Lpg0085 the DD-link is a single residue (Figure 7A,B; Table 1 and Table S1, rows 18c and 18d). To compensate for such a reduction in the DD-link length in Lpg0085, a mediator dipeptide Arg_147_-Asp_148_ is additionally present for DD-zone formation. Thus, the DD-zones of the dimeric aspartyl proteases and the Lpg0085-like proteins are characterized by the presence of either a longer DD-link of four residues or a shorter DD-link of one residue plus a separate two-residue mediator.

**Figure 8:**
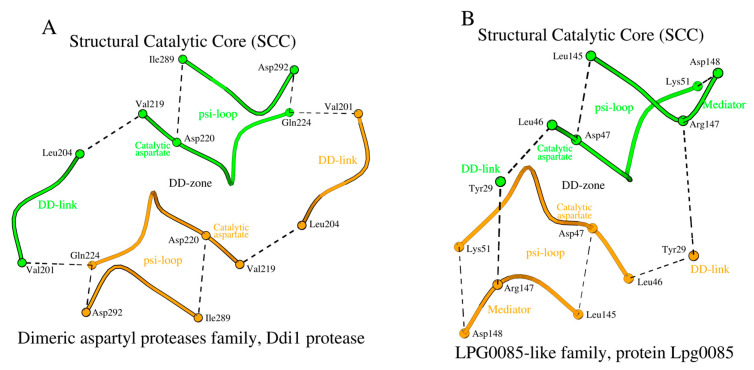
SCC of (**A**) Ddi1 protease and (**B**) protein Lpg0085. The main differences between the SCCs of the two proteins are the amino acid composition of the DD-links and the use of a mediator-dipeptide in the structural formation of the DD-zone in the protein Lpg0085.

As in the case of retroviral proteases, Ddi1 and Lpg0085 use the psi-loop_C_ motif, which is equivalent to the C-terminal version of the psi-loop motif in pepsin-like family proteins (Table 1 and Table S2, rows 16c, 16d, 18c and 18d). The ApRick protease does not form a canonical dimer, as do Ddi1 and Lpg0085 [25]. However, the psi-loop in the ApRick protease monomer is still identical to that in Ddi1 and Lpg0085 (Figure 5C; Table 1 and Table S2, row 17c). Li et al. suggested that the ApRick protease “may represent a putative common ancestor of monomeric and dimeric aspartic proteases” [25]. The SCCs in Ddi1 and Lpg0085 are shown in Figure 8A,B.

## 3. Conclusions

Here, we have outlined the minimal conserved structural arrangement common to the acid protease superfamily of proteins, which we refer to as the structural catalytic core (SCC). We began with the pepsin-like family proteases, where we defined the DD-zone (Figure 1A). The DD-zone is a circular structural motif defined by substructures around the catalytic aspartates in the N- and C-terminal domains, D-loop_N_ and D-loop_C_, and their interactions with the peptides DD-link_N_ and DD-link_C_, which join the ends of D-loop_N_ and D-loop_C_. Then, we increased the common substructure by defining the psi-loop_N_ and psi-loop_C_ motifs, where the DD-zone interacts through their D-loops with two external tetrapeptides, G-loop_N_ and G-loop_C_, the residues of which intersect with the Hydrophobic-Hydrophobic-Gly sequence motif [51] (Figure 1B,C). While the two psi-loop motifs use the same logic in their formation, they differ in the environment around the catalytic aspartates, which may determine their different functional roles. Taken together, the psi-loops and the DD-zone define structural boundaries of the SCC in pepsin-like proteins.

The other families of acid proteases, retroviral proteases (retropepsin), dimeric aspartyl proteases, and Lpg0085-like proteins, also have the DD-zone and psi-loop substructures similar to pepsin. However, unlike pepsin—which can be very roughly described as a “hetero psi-loop” protein, where psi-loop_N_ and psi-loop_C_ are not structurally identical unlike the homodimer enzymes, with the psi-loop_C_ being more functionally active—the retroviral proteases, dimeric aspartyl proteases, and Lpg0085-like proteins can be described as having a “homo psi-loop” since they have two identical chains. The homo psi-loops are both structurally similar to psi-loop_C_ of pepsin. As with the pepsin-like proteases, the other three protein families use DD-links to form a DD-zone (Table 1). If a DD-link is equal to or shorter than two amino acids, there are additional mediator residues or water molecules filling the gap. Some mediator residues are located in sequence either at the C-terminus of the G-loop or immediately after it. Based on the structures seen so far, we can argue that a specific “long DD-link”, or “DD-link + mediator” or “DD-link + water” combination, is the same for a structural family within an acid protease superfamily, and may distinguish that family from the other proteins.

In summary, we can say that the SCC of the acid protease superfamily proteins consists of a dimer composed of a DD-link, D-loop, and G-loop blocks, where the D-loop plus DD-link forms a DD-zone, and the dimer of D- and G-loops forms two psi-loops. Defining the SCC in this way allows us to outline a minimal common substructure for the entire superfamily of proteins, such as acid proteases. This substructure combines amino acid conservation and protein functionality, which together can be used for protein comparison, structure identification, protein family separation, and protein engineering.

## Data Availability

All data supporting reported results can be found in Supplementary Materials.

## Supplementary Materials

The following supporting information can be downloaded at: https://www.mdpi.com/article/10.3390/molecules29153451/s1, Table S1. Conserved geometric parameters (distance and angle) of contacts in 33 DD-zones of the acid proteases superfamily proteins. Table S2. Conserved geometric parameters (distance and angle) of contacts in 65 psi-loops of the acid protease superfamily proteins and contacts between DD-link_N_ and the propeptide/N-terminal peptide in 13 pepsin-like family proteins. Table S3. Conserved geometric parameters (distance and angle) of contacts between hydrolase and ligand in nine acid protease pepsin-like and retroviral protease (retropepsin) families.
