# Supplementary material for: Structural Catalytic Core of the Members of the Superfamily of Acid Proteases
Source: Molecules. 2024 Jul 23;29(15):3451. doi: 10.3390/molecules29153451 (PMC11313796; doi:10.3390/molecules29153451)
Supplement: Supplementary file 1 [file molecules-29-03451-s001.zip › molecules-3093973-supplementary.pdf]

Supplementary materials

Table S1. Conserved geometric parameters (distance and angle) of contacts in 33 DD-zones of the acid proteases superfamily proteins.

N	PDB ID	Protein	DD-zone (Circular DD-links & D-loops connection)				Fireman's grip
			Superfamily: Acid proteases				
			Family: Pepsin-like				
1a, b	3PSG_A	Propepsin	O/Y$_{14}$-HOH$_{507}$ 2.7<br>HOH$_{507}$-N/F$_{31}$ 2.8	OG/S$_{36}$-O/Y$_{125}$ 2.7<br>N/Y$_{125}$-O/G$_{188}$ 3.2	O/W$_{190}$-N/V$_{214}$ 2.9	OG/S$_{219}$-O/D$_{11}$ 2.9	O/F$_{31}$-OG1/T$_{216}$ 2.7<br>N/T$_{33}$-OG1/T$_{216}$ 2.8<br>OG1/T$_{33}$-N/T$_{216}$ 2.9<br>OG1/T$_{33}$-O/V$_{214}$ 3.1
1c, d	4PEP_A	Pepsin	O/Y$_{14}$-HOH$_{426}$ 3.2<br>HOH$_{426}$-N/F$_{31}$ 3.1	OG/S$_{36}$-O/Y$_{125}$ 2.6<br>N/Y$_{125}$-O/G$_{188}$ 2.8	O/W$_{190}$-N/V$_{214}$ 2.9	OG/S$_{219}$-O/T$_{12}$ 3.0	O/F$_{31}$-OG1/T$_{216}$ 2.6<br>N/T$_{33}$-OG1/T$_{216}$ 3.0<br>OG1/T$_{33}$-N/T$_{216}$ 3.0<br>OG1/T$_{33}$-O/V$_{214}$ 2.9
1e, f	6XCZ_A	Pepsin	O/Y$_{14}$-N/F$_{31}$ 3.8	OG/S$_{36}$-O/Y$_{125}$ 2.7<br>N/Y$_{125}$-O/G$_{188}$ 2.9	O/W$_{190}$-N/V$_{214}$ 3.0	OG/S$_{219}$-O/T$_{12}$ 2.4	O/F$_{31}$-OG1/T$_{216}$ 2.6<br>N/T$_{33}$-OG1/T$_{216}$ 3.0<br>OG1/T$_{33}$-N/T$_{216}$ 2.9<br>OG1/T$_{33}$-O/V$_{214}$ 2.8
2a, b	3VCM_A	Prorenin	O/Y$_{14}$-N/F$_{31}$ 5.2	OG/S$_{36}$-O/F$_{125}$ 2.7<br>N/F$_{125}$-O/G$_{188}$ 4.2	O/W$_{190}$-N/V$_{214}$ 3.0	CB/S$_{219}$-O/D$_{11}$<br>3.2 (2.5) 120°	O/F$_{31}$-OG1/T$_{216}$ 2.6<br>N/T$_{33}$-OG1/T$_{216}$ 2.9<br>OG1/T$_{33}$-N/T$_{216}$ 2.9<br>OG1/T$_{33}$-O/V$_{214}$ 2.7
2c, d	2REN_A	Renin	O/Y$_{20}$-N/F$_{37}$ 4.0	OG/S$_{42}$-O/F$_{132}$ 3.2<br>N/F$_{132}$-O/G$_{199}$ 4.3	O/W$_{201}$-N/V$_{225}$ 3.0	OG/S$_{230}$-O/T$_{18}$ 3.3	O/F$_{37}$-OG1/T$_{227}$ 2.7<br>N/T$_{39}$-OG1/T$_{227}$ 2.8<br>OG1/T$_{39}$-N/T$_{227}$ 2.9<br>OG1/T$_{39}$-O/V$_{225}$ 2.6
2e, f	3K1W_A	Renin	O/Y$_{20}$-HOH$_{357}$ 2.8<br>HOH$_{357}$-N/F$_{37}$ 2.9	OG/S$_{42}$-O/F$_{132}$ 2.7<br>N/F$_{132}$-HOH$_{387}$ 3.0<br>HOH$_{387}$-O/G$_{199}$ 2.9	O/W$_{201}$-N/V$_{225}$ 2.9	OG/S$_{230}$-O/T$_{18}$ 2.7	O/F$_{37}$-OG1/T$_{227}$ 2.7<br>N/T$_{39}$-OG1/T$_{227}$ 2.9<br>OG1/T$_{39}$-N/T$_{227}$ 2.8<br>OG1/T$_{39}$-O/V$_{225}$ 2.7
3a, b	1PFZ_A	Proplasmepsin 2	O/M$_{15}$-N/L$_{33}$ 2.9	CB/A$_{38}$-O/W$_{128}$<br>3.0 (2.0) 169°<br>N/W$_{128}$-HOH$_{469}$ 2.8<br>HOH$_{469}$-O/L$_{191}$ 2.6	O/W$_{193}$-N/V$_{213}$ 3.0	OG/S$_{218}$-O/Q$_{12}$ 3.1	O/L$_{33}$-OG/S$_{215}$ 6.4<br>N/T$_{35}$-OG/S$_{215}$ 6.0<br>OG1/T$_{35}$-N/S$_{215}$ 4.9<br>OG1/T$_{35}$-O/V$_{213}$ 4.5
3c, d	1LF4_A	Plasmepsin 2	O/F$_{16}$-HOH$_{339}$ 2.7<br>HOH$_{339}$-N/L$_{33}$ 2.8	CB/A$_{38}$-O/W$_{128}$<br>3.3 (2.7) 113°<br>N/W$_{128}$-O/L$_{191}$ 3.7	O/W$_{193}$-N/V$_{213}$ 3.1	CB/S$_{218}$-O/I$_{14}$<br>3.2 (2.6) 114°	O/L$_{33}$-OG/S$_{215}$ 2.6<br>N/T$_{35}$-OG/S$_{215}$ 3.0<br>OG1/T$_{35}$-N/S$_{215}$ 2.9<br>OG1/T$_{35}$-O/V$_{213}$ 3.3
3e, f	2BJU_A	Plasmepsin 2	O/F$_{16}$-HOH$_{2051}$ 2.7<br>HOH$_{2051}$-N/L$_{33}$ 2.9	CB/A$_{38}$-O/W$_{128}$<br>3.4 (3.0) 104°<br>N/W$_{128}$-O/L$_{191}$ 3.1	O/W$_{193}$-N/V$_{213}$ 2.9	CB/S$_{218}$-O/I$_{14}$<br>2.9 (2.2) 115°	O/L$_{33}$-OG/S$_{215}$ 2.6<br>N/T$_{35}$-OG/S$_{215}$ 2.9<br>OG1/T$_{35}$-N/S$_{215}$ 2.9<br>OG1/T$_{35}$-O/V$_{213}$ 2.9
4a, b	3QVC_A	HAP zymogen	O/L$_{13}$-N/F$_{31}$ 2.8	OG/S$_{36}$-O/W$_{125}$ 3.7<br>N/W$_{125}$-HOH$_{398}$ 2.9<br>HOH$_{398}$-O/L$_{188}$ 2.5	O/W$_{190}$-N/L$_{214}$ 2.8	OG/S$_{219}$-O/L$_{9}$ 2.9	O/F$_{31}$-OG/S$_{216}$ 7.7<br>N/T$_{33}$-OG/S$_{216}$ 7.3<br>OG1/T$_{33}$-N/S$_{216}$ 5.0<br>OG1/T$_{33}$-HOH$_{430}$ 2.9<br>HOH$_{430}$-O/L$_{214}$ 2.9
4e, f	3QVI_A	HAP protein	O/S$_{14}$-N/F$_{31}$ 2.9	OG/S$_{36}$-O/W$_{125}$ 2.6<br>N/W$_{125}$-HOH$_{590}$ 2.5<br>HOH$_{590}$-O/L$_{188}$ 2.6	O/W$_{190}$-N/L$_{214}$ 2.9	OG/S$_{219}$-CD1/L$_{13}$<br>3.8 (3.2) 113°<br>CD2/L$_{13}$-O/V$_{12}$<br>2.8 (1.8) 156°	O/F$_{31}$-OG/S$_{216}$ 2.9<br>N/T$_{33}$-OG/S$_{216}$ 2.8<br>OG1/T$_{33}$-N/S$_{216}$ 2.9<br>OG1/T$_{33}$-O/L$_{214}$ 3.1
5a, b	5N7N_A	Procathepsin D	O/Y$_{40}$-N/F$_{57}$ 4.1	CB/A$_{62}$-O/Y$_{151}$<br>3.3 (2.7) 113°<br>N/Y$_{151}$-O/G$_{217}$ 2.9	O/W$_{219}$-N/A$_{248}$ 3.1	OG/S$_{253}$-O/D$_{37}$ 4.1	O/F$_{57}$-OG1/T$_{250}$ 2.8<br>N/T$_{59}$-OG1/T$_{250}$ 2.9<br>OG1/T$_{59}$-N/T$_{250}$ 2.9<br>OG1/T$_{59}$-O/A$_{248}$ 2.9
5c, d	5N71_A	Cathepsin D	O/Y$_{40}$-HOH$_{565}$ 2.8<br>HOH$_{565}$-N/F$_{57}$ 2.8	CB/A$_{62}$-O/Y$_{151}$<br>3.5 (2.8) 117°<br>N/Y$_{151}$-O/G$_{217}$ 3.0	O/W$_{219}$-N/A$_{248}$ 3.2	OG/S$_{253}$-O/V$_{38}$ 3.4	O/F$_{57}$-OG1/T$_{250}$ 2.6<br>N/T$_{59}$-OG1/T$_{250}$ 2.8<br>OG1/T$_{59}$-N/T$_{250}$ 2.9<br>OG1/T$_{59}$-O/A$_{248}$ 2.8
5e, f	5N7Q_A	Cathepsin D	O/Y$_{18}$-HOH$_{595}$ 2.7	CB/A$_{40}$-O/Y$_{129}$	O/W$_{197}$-N/A$_{226}$ 3.0	OG/S$_{231}$-O/V$_{16}$ 2.6	O/F$_{35}$-OG1/T$_{228}$ 2.7

			HOH$_{595}$-N/F$_{35}$ 2.8	3.4 (2.7) 124°<br>N/Y$_{129}$-O/G$_{195}$ 3.0			N/T$_{37}$-OG1/T$_{228}$ 2.9<br>OG1/T$_{37}$-N/T$_{228}$ 2.9<br>OG1/T$_{37}$-O/A$_{226}$ 2.8
6a, b	1MIQ_A	Proplasmepsin	O/M$_{15}$-N/F$_{33}$ 3.1	CB/A$_{38}$-O/W$_{128}$<br>3.5 (2.5) 147°<br>N/W$_{128}$-HOH$_{362}$ 2.7<br>HOH$_{362}$-O/L$_{191}$ 2.8	O/W$_{193}$-N/V$_{213}$ 3.1	OG1/T$_{218}$-ND2/N$_{13}$ 3.4	O/F$_{33}$-OG/S$_{215}$ 7.0<br>N/T$_{35}$-OG/S$_{215}$ 6.7<br>OG1/T$_{35}$-N/S$_{215}$ 5.1<br>OG1/T$_{35}$-HOH$_{342}$ 2.7<br>HOH$_{342}$-O/V$_{213}$ 3.0
6e, f	1QS8_A	Plasmepsin	O/F$_{16}$-HOH$_{350}$ 3.1<br>HOH$_{350}$-N/F$_{33}$ 2.7	CB/A$_{38}$-O/W$_{128}$<br>3.2 (2.7) 105°<br>N/W$_{128}$-O/L$_{191}$ 3.1	O/W$_{193}$-N/V$_{213}$ 2.9	CG2/T$_{218}$-O/I$_{14}$<br>4.0 (3.4) 114°	O/F$_{33}$-OG/S$_{215}$ 2.5<br>N/T$_{35}$-OG/S$_{215}$ 3.0<br>OG1/T$_{35}$-N/S$_{215}$ 3.0<br>OG1/T$_{35}$-O/V$_{213}$ 2.7
7a, b	5JOD_A	Proplasmepsin 4	O/M$_{15}$-N/F$_{33}$ 3.1	CB/A$_{38}$-O/W$_{128}$<br>4.3 (3.6) 122°<br>N/W$_{128}$-HOH$_{513}$ 2.9<br>HOH$_{513}$-O/L$_{191}$ 2.5	O/W$_{193}$-N/V$_{213}$ 3.0	CB/S$_{218}$-OD1/N$_{13}$<br>3.3 (2.7) 118°	O/F$_{33}$-OG/S$_{215}$ 7.4<br>N/T$_{35}$-OG/S$_{215}$ 6.9<br>OG1/T$_{35}$-N/S$_{215}$ 5.1<br>OG1/T$_{35}$-HOH$_{681}$ 3.0<br>HOH$_{681}$-O/V$_{213}$ 2.9
7e, f	1LS5_A	Plasmepsin 4	O/F$_{16}$-N/F$_{33}$ 4.8	CB/A$_{38}$-O/W$_{128}$<br>3.6 (3.3) 98°<br>N/W$_{128}$-O/L$_{191}$ 3.0	O/W$_{193}$-N/V$_{213}$ 3.1	CB/S$_{218}$-O/L$_{14}$<br>2.9 (2.1) 123°	O/F$_{33}$-OG/S$_{215}$ 2.9<br>N/T$_{35}$-OG/S$_{215}$ 2.8<br>OG1/T$_{35}$-N/S$_{215}$ 3.2<br>OG1/T$_{35}$-O/V$_{213}$ 2.9
8a, b	1QDM_A	Prophytepsin	O/Y$_{18}$-N/F$_{35}$ 4.9	OG/S$_{40}$-O/F$_{130}$ 2.5<br>N/F$_{130}$-O/G$_{195}$ 3.5	O/W$_{197}$-N/A$_{222}$ 3.1	OG/S$_{227}$-O/N$_{15}$ 2.8	O/F$_{35}$-OG/S$_{224}$ 3.6<br>N/T$_{37}$-OG/S$_{224}$ 3.4<br>OG1/T$_{37}$-N/S$_{224}$ 2.9<br>OG1/T$_{37}$-O/A$_{222}$ 3.0
9a, b	1HTR_B	Progastricsin	O/Y$_{14}$-HOH$_{378}$ 2.7<br>HOH$_{378}$-N/F$_{31}$ 2.8	CB/S$_{36}$-CB/Y$_{125}$ 4.9<br>CA/Y$_{125}$-O/L$_{189}$<br>3.9 (2.8) 161°	O/W$_{191}$-N/V$_{216}$ 2.9	OG/S$_{221}$-HOH$_{396}$ 3.1<br>HOH$_{396}$-O/D$_{11}$ 2.9	O/F$_{31}$-OG1/T$_{218}$ 2.7<br>N/T$_{33}$-OG1/T$_{218}$ 3.0<br>OG1/T$_{33}$-N/T$_{218}$ 2.8<br>OG1/T$_{33}$-O/V$_{216}$ 2.8
10a, b	1TZS_A	Procathepsin E	O/Y$_{25}$-HOH$_{388}$ 2.6<br>HOH$_{388}$-N/F$_{42}$ 2.9	OG/S$_{47}$-O/Y$_{136}$ 2.6<br>N/Y$_{136}$-O/A$_{201}$ 2.9	O/W$_{203}$-N/V$_{227}$ 3.1	OG/S$_{232}$-O/M$_{23}$ 2.6<br>OG/S$_{232}$-O/D$_{22}$ 3.3	O/F$_{42}$-OG1/T$_{229}$ 2.6<br>N/T$_{44}$-OG1/T$_{229}$ 3.0<br>OG1/T$_{44}$-N/T$_{229}$ 3.0<br>OG1/T$_{44}$-O/V$_{227}$ 2.8
11c, d	1T6E_X	Xylanase inhibitor	O/Y$_{16}$-N/L$_{28}$ 2.8	O/P$_{33}$-OG/S$_{146}$ 2.7<br>N/N$_{145}$-O/P$_{204}$ 2.7	O/H$_{206}$-N/L$_{234}$ 3.0	CG/P$_{239}$-CB/S$_{14}$ 4.4	O/L$_{28}$-OG1/T$_{236}$ 2.8<br>N/V$_{30}$-OG1/T$_{236}$ 3.1<br>CG2/V$_{30}$-N/T$_{236}$ 3.6<br>CG2/V$_{30}$-O/L$_{234}$<br>3.4 (2.6) 130°
11e, f	1T6G_A	Xylanase inhibitor	O/Y$_{16}$-N/L$_{28}$ 2.8	O/P$_{33}$-OG/S$_{146}$ 3.5<br>N/N$_{145}$-O/P$_{204}$ 2.9	O/H$_{206}$-N/L$_{234}$ 3.1	CG/P$_{239}$-CB/S$_{14}$ 4.3	O/L$_{28}$-OG1/T$_{236}$ 2.7<br>N/V$_{30}$-OG1/T$_{236}$ 3.0<br>CG2/V$_{30}$-N/T$_{236}$ 3.7<br>CG2/V$_{30}$-O/L$_{234}$<br>3.4 (2.6) 133°
12c, d	3AUP_A	Basic 7S globulin	O/H$_{23}$-N/V$_{40}$ 2.8	OD1/N$_{45}$-CB/A$_{164}$<br>3.2 (2.6) 112°<br>N/H$_{163}$-HOH$_{512}$ 3.0<br>HOH$_{512}$-O/G$_{228}$ 2.7	O/Y$_{230}$-N/I$_{264}$ 3.2	CB/P$_{269}$-O/G$_{21}$<br>4.0 (3.0) 146°	O/V$_{40}$-OG1/T$_{266}$ 2.6<br>N/L$_{42}$-OG1/T$_{266}$ 3.0<br>CB/L$_{42}$-N/T$_{266}$ 3.9<br>CD2/L$_{42}$-O/I$_{264}$<br>3.4 (2.3) 175°
13c, d	3VLA_A	EDGP (Fragment)	O/Y$_{22}$-N/V$_{39}$ 2.8	O/R$_{44}$-OG1/T$_{160}$ 2.7<br>N/R$_{159}$-O/V$_{235}$ 2.9	O/Y$_{237}$-N/I$_{270}$ 3.1	CB/P$_{275}$-O/L$_{20}$<br>4.4 (3.4) 158°	O/V$_{39}$-OG1/T$_{272}$ 2.8<br>N/L$_{41}$-OG1/T$_{272}$ 3.0<br>CB/L$_{41}$-N/T$_{272}$ 4.2<br>CD2/L$_{41}$-O/I$_{270}$<br>3.4 (2.4) 156°
13e, f	3VLB_A	EDGP (Fragment)	O/Y$_{22}$-N/V$_{39}$ 3.0	O/R$_{44}$-OG1/T$_{160}$ 2.8<br>N/R$_{159}$-O/V$_{235}$ 3.1	O/Y$_{237}$-N/I$_{270}$ 2.9	CB/P$_{275}$-O/L$_{20}$<br>4.2 (3.2) 152°	O/V$_{39}$-OG1/T$_{272}$ 2.5<br>N/L$_{41}$-OG1/T$_{272}$ 3.3<br>CB/L$_{41}$-N/T$_{272}$ 4.4<br>CD2/L$_{41}$-O/I$_{270}$<br>3.6 (2.6) 146°
				Family: Retroviral protease (retropepsin)			
14c, d	3IXO_A, B	HIV-1 protease	O/P$_{9_A}$-N/L$_{24_A}$ 2.8	OD1/D$_{29_A}$-NH2/R$_{87_A}$ 2.5<br>OD1/D$_{29_A}$-NE/R$_{8_B}$ 3.3<br>OD2/D$_{29_A}$-NH2/R$_{8_B}$ 3.3	O/P$_{9_B}$-N/L$_{24_B}$ 3.0	OD1/D$_{29_B}$-NH2/R$_{87_B}$ 3.5<br>OD1/D$_{29_B}$-NE/R$_{8_A}$ 3.2<br>OD2/D$_{29_B}$-NH2/R$_{8_A}$ 2.8	O/L$_{24_A}$-OG1/T$_{26_B}$ 2.9<br>N/T$_{26_A}$-OG1/T$_{26_B}$ 2.9<br>OG1/T$_{26_A}$-N/T$_{26_B}$ 2.9

							OG1/T$_{26_A}$-O/L$_{24_B}$ 2.5
14e, f	5YOK_A, B	HIV-1 protease	O/P$_{9_A}$-N/L$_{24_A}$ 2.9	OD1/D$_{29_A}$-NH2/R$_{87_A}$ 2.9<br>OD1/D$_{29_A}$-NE/R$_{8_B}$ 3.0<br>OD2/D$_{29_A}$-NH2/R$_{8_B}$ 2.8	O/P$_{9_B}$-N/L$_{24_B}$ 2.9	OD1/D$_{29_B}$-NH2/R$_{87_B}$ 2.9<br>OD1/D$_{29_B}$-NE/R$_{8_A}$ 2.8<br>OD2/D$_{29_B}$-NH2/R$_{8_A}$ 2.9	O/L$_{24_A}$-OG1/T$_{26_B}$ 2.7<br>N/T$_{26_A}$-OG1/T$_{26_B}$ 2.9<br>OG1/T$_{26_A}$-N/T$_{26_B}$ 2.9<br>OG1/T$_{26_A}$-O/L$_{24_B}$ 2.7
15c, d	3NR6_A, B	XMRV protease	O/P$_{16_A}$-N/V$_{31_A}$ 2.9	OE1/Q$_{36_A}$-NH2/R$_{95_A}$ 5.0<br>CD/R$_{95_A}$-O/E$_{15_B}$<br>3.1 (2.6) 108°	O/P$_{16_B}$-N/V$_{31_B}$ 2.9	OE1/Q$_{36_B}$-CB/R$_{95_B}$<br>3.9 (3.0) 150°<br>CD/R$_{95_B}$-O/E$_{15_A}$<br>3.0 (2.6) 104°	O/V$_{31_A}$-OG1/T$_{33_B}$ 2.6<br>N/T$_{33_A}$-OG1/T$_{33_B}$ 3.0<br>OG1/T$_{33_A}$-N/T$_{33_B}$ 2.9<br>OG1/T$_{33_A}$-O/V$_{31_B}$ 2.6
15e, f	3SLZ_A, B	XMRV protease	O/P$_{16_A}$-N/V$_{31_A}$ 2.8	OE1/Q$_{36_A}$-HOH$_{178}$ 3.0<br>HOH$_{178}$-NH2/R$_{95_A}$ 2.9<br>CD/R$_{95_A}$-O/E$_{15_B}$<br>3.3 (2.8) 108°	O/P$_{16_B}$-N/V$_{31_B}$ 2.9	OE1/Q$_{36_B}$-NH2/R$_{95_B}$ 4.6<br>CD/R$_{95_B}$-O/E$_{15_A}$<br>3.2 (2.6) 114°	O/V$_{31_A}$-OG1/T$_{33_B}$ 2.6<br>N/T$_{33_A}$-OG1/T$_{33_B}$ 2.9<br>OG1/T$_{33_A}$-N/T$_{33_B}$ 2.9<br>OG1/T$_{33_A}$-O/V$_{31_B}$ 2.7
				Family: Dimeric aspartyl proteases			
16c, d	4Z2Z_A, B	Ddi1 protease	O/L$_{204_A}$-N/V$_{219_A}$ 2.8	NE2/Q$_{224_A}$-O/V$_{201_B}$ 3.4	O/L$_{204_B}$-N/V$_{219_B}$ 2.8	NE2/Q$_{224_B}$-O/V$_{201_A}$ 4.2	O/V$_{219_A}$-OG/T$_{221_B}$ 3.0<br>N/T$_{221_A}$-OG1/T$_{221_B}$ 2.9<br>OG1/T$_{221_A}$-N/T$_{221_B}$ 2.8<br>OG1/T$_{221_A}$-N/V$_{219_B}$ 2.9
17c	5C9F_A	ApRick protease	O/F$_{124}$-N/V$_{139}$ 2.9	N/A	N/A	N/A	N/A
				Family: LPG0085-like			
18c, d	2PMA_A, I	Protein Lpg0085	O/Y$_{29_A}$-N/L$_{46_A}$ 2.8	NZ/K$_{51_A}$-OD1/D$_{148_A}$ 3.0<br>R$_{147_A}$-Y$_{29_I}$ 3.9 (π-π)	O/Y$_{29_I}$-N/L$_{46_I}$ 2.7	NZ/K$_{51_I}$-OD2/D$_{148_I}$ 3.2<br>R$_{147_I}$-Y$_{29_A}$ 3.9 (π-π)	O/L$_{46_A}$-OG1/T$_{48_I}$ 2.7<br>N/T$_{48_A}$-OG1/T$_{48_I}$ 2.9<br>OG1/T$_{48_A}$-N/T$_{48_I}$ 3.0<br>OG1/T$_{48_A}$-O/L$_{46_I}$ 2.7

N/A – Not Available.

Table S2. Conserved geometric parameters (distance and angle) of contacts in 65 psi-loops of the acid proteases superfamily proteins and contacts between DD-link$_N$ and the propeptide/N-terminal peptide in 13 pepsin-like family proteins.

N	PDB ID	Protein	psi-loop (D-loop & G-loop)		Asx-motif	DD-link$_N$/Propep. DD-link$_N$/N-pep.
			Superfamily: Acid proteases			
			Family: Pepsin-like			
1a	3PSG_A, p	Propepsin	N/D$_{32}$-O/L$_{121}$ 3.1<br>O/D$_{32}$-N/L$_{123}$ 2.8<br>O/S$_{35}$-N/A$_{124}$ 2.7	O/D$_{32}$-CA/G$_{122}$<br>3.5 (2.6) 143°	OD1/D$_{32}$-N/G$_{34}$ 2.8<br>O/D$_{32}$-N/S$_{35}$ 3.3	O/E$_{13}$-N/K$_{9P}$ 2.8<br>N/F$_{15}$-O/V$_{7P}$ 3.0<br>O/F$_{15}$-N/V$_{7P}$ 2.7
1b	3PSG_A	Propepsin	N/D$_{215}$-O/L$_{301}$ 3.0<br>O/T$_{218}$-N/D$_{303}$ 2.9<br>O/S$_{219}$-N/V$_{304}$ 3.1	O/D$_{215}$-CA/G$_{302}$<br>3.4 (2.4) 157°<br>O/G$_{302}$-N/F$_{305}$ 3.0	OD1/D$_{215}$-N/G$_{217}$<br>2.6<br>O/D$_{215}$-N/T$_{218}$ 3.4	
1c	4PEP_A	Pepsin	N/D$_{32}$-O/L$_{121}$ 2.9<br>O/D$_{32}$-N/L$_{123}$ 2.9<br>O/S$_{35}$-N/A$_{124}$ 2.8	O/D$_{32}$-CA/G$_{122}$<br>3.5 (2.6) 141°	OD1/D$_{32}$-N/G$_{34}$ 3.1<br>O/D$_{32}$-N/S$_{35}$ 3.4	O/E$_{13}$-N/Y$_{9}$ 2.8<br>N/F$_{15}$-O/E$_{7}$ 3.2<br>O/F$_{15}$-N/E$_{7}$ 2.7
1d	4PEP_A	Pepsin	N/D$_{215}$-O/L$_{301}$ 2.9<br>O/T$_{218}$-N/D$_{303}$ 2.7	O/D$_{215}$-CA/G$_{302}$<br>3.5 (2.4) 163°	OD2/D$_{215}$-N/G$_{217}$<br>3.0	

			O/S$_{219}$-N/V$_{304}$ 3.0	O/G$_{302}$-N/F$_{305}$ 3.0	O/D$_{215}$-N/T$_{218}$ 3.3	
1e	6XCZ_A	Pepsin	N/D$_{32}$-O/L$_{121}$ 2.9<br>O/D$_{32}$-N/L$_{123}$ 2.9<br>O/S$_{35}$-N/A$_{124}$ 3.0	O/D$_{32}$-CA/G$_{122}$<br>3.4 (2.5) 146°	OD1/D$_{32}$-N/G$_{34}$ 3.0<br>O/D$_{32}$-N/S$_{35}$ 3.4	O/E$_{13}$-N/Y$_{9}$ 3.0<br>N/F$_{15}$-O/E$_{7}$ 3.3<br>O/F$_{15}$-N/E$_{7}$ 2.8
1f	6XCZ_A	Pepsin	N/D$_{215}$-O/L$_{301}$ 3.1<br>O/T$_{218}$-N/D$_{303}$ 2.8<br>O/S$_{219}$-N/V$_{304}$ 3.2	O/D$_{215}$-CA/G$_{302}$<br>3.5 (2.4) 162°<br>O/G$_{302}$-N/F$_{305}$ 2.9	OD1/D$_{215}$-N/G$_{217}$<br>2.9<br>O/D$_{215}$-N/T$_{218}$ 3.2	
2a	3VCM_A, p	Prorenin	N/D$_{32}$-O/V$_{121}$ 3.1<br>O/D$_{32}$-N/M$_{123}$ 2.7<br>O/S$_{35}$-N/G$_{124}$ 2.8	O/D$_{32}$-CA/G$_{122}$<br>3.5 (2.6) 139°	OD1/D$_{32}$-N/G$_{34}$ 2.9<br>O/D$_{32}$-N/S$_{35}$ 3.7	O/Q$_{13}$-N/M$_{16P}$ 2.7<br>N/Y$_{15}$-O/K$_{14P}$ 3.1<br>O/Y$_{15}$-N/K$_{14P}$ 2.9
2b	3VCM_A	Prorenin	N/D$_{215}$-O/L$_{301}$ 3.2<br>O/A$_{218}$-N/A$_{303}$ 2.5<br>O/S$_{219}$-N/T$_{304}$ 3.2	O/D$_{215}$-CA/G$_{302}$<br>3.3 (2.2) 170°<br>O/G$_{302}$-N/F$_{305}$ 3.1	OD1/D$_{215}$-N/G$_{217}$<br>3.0<br>O/D$_{215}$-N/A$_{218}$ 3.5	
2c	2REN_A	Renin	N/D$_{38}$-O/V$_{128}$ 3.1<br>O/D$_{38}$-N/M$_{130}$ 2.8<br>O/S$_{41}$-N/G$_{131}$ 2.9	O/D$_{38}$-CA/G$_{129}$<br>3.4 (2.6) 125°	OD1/D$_{38}$-N/G$_{40}$ 2.8<br>O/D$_{38}$-N/S$_{41}$ 3.0	O/Q$_{19}$-N/Y$_{15}$ 2.6<br>N/Y$_{21}$-O/T$_{13}$ 2.9<br>O/Y$_{21}$-N/T$_{13}$ 2.9
2d	2REN_A	Renin	N/D$_{226}$-O/L$_{315}$ 2.9<br>O/A$_{229}$-N/A$_{317}$ 2.5<br>O/S$_{230}$-N/T$_{318}$ 3.5	O/D$_{226}$-CA/G$_{316}$<br>2.9 (2.1) 153°<br>O/G$_{316}$-N/F$_{319}$ 3.0	OD1/D$_{226}$-N/G$_{228}$<br>3.3<br>O/D$_{226}$-N/A$_{229}$ 3.3	
2e	3K1W_A	Renin	N/D$_{38}$-O/V$_{128}$ 2.9<br>O/D$_{38}$-N/M$_{130}$ 2.8<br>O/S$_{41}$-N/G$_{131}$ 2.8	O/D$_{38}$-CA/G$_{129}$<br>3.5 (2.5) 141°	OD1/D$_{38}$-N/G$_{40}$ 3.1<br>O/D$_{38}$-N/S$_{41}$ 3.4	O/Q$_{19}$-N/Y$_{15}$ 2.8<br>N/Y$_{21}$-O/T$_{13}$ 3.1<br>O/Y$_{21}$-N/T$_{13}$ 2.8
2f	3K1W_A	Renin	N/D$_{226}$-O/L$_{315}$ 2.9<br>O/A$_{229}$-N/A$_{317}$ 2.9<br>O/S$_{230}$-N/T$_{318}$ 3.4	O/D$_{226}$-CA/G$_{316}$<br>3.3 (2.2) 155°<br>O/G$_{316}$-N/F$_{319}$ 2.9	OD1/D$_{226}$-N/G$_{228}$<br>3.0<br>O/D$_{226}$-N/A$_{229}$ 3.1	
3a	1PFZ_A, p	Proplasmepsin 2	N/D$_{34}$-O/L$_{124}$ 3.1<br>O/D$_{34}$-N/L$_{126}$ 2.9<br>O/S$_{37}$-N/G$_{127}$ 2.8	O/D$_{34}$-CA/G$_{125}$<br>3.6 (2.6) 144°	OD1/D$_{34}$-N/G$_{36}$ 2.7<br>O/D$_{34}$-N/S$_{37}$ 3.3	O/I$_{14}$-N/E$_{87P}$ 2.9<br>N/F$_{16}$-O/K$_{85P}$ 2.8<br>O/F$_{16}$-N/K$_{85P}$ 3.0
3b	1PFZ_A	Proplasmepsin 2	N/D$_{214}$-O/L$_{301}$ 3.1<br>O/T$_{217}$-N/D$_{303}$ 2.8<br>O/S$_{218}$-CD/P$_{304}$<br>3.4 (2.6) 131°	O/D$_{214}$-CA/G$_{302}$<br>3.2 (2.2) 156°<br>O/G$_{302}$-N/F$_{305}$ 3.0	OD1/D$_{214}$-N/G$_{216}$<br>2.8<br>O/D$_{214}$-N/T$_{217}$ 3.2	
3c	1LF4_A	Plasmepsin 2	N/D$_{34}$-O/L$_{124}$ 3.0<br>O/D$_{34}$-N/L$_{126}$ 2.7<br>O/S$_{37}$-N/G$_{127}$ 2.8	O/D$_{34}$-CA/G$_{125}$<br>3.4 (2.5) 139°	OD2/D$_{34}$-N/G$_{36}$ 2.8<br>O/D$_{34}$-N/S$_{37}$ 3.3	O/M$_{15}$-N/F$_{11}$ 2.7<br>N/Y$_{17}$-O/V$_{9}$ 2.8<br>O/Y$_{17}$-N/V$_{9}$ 3.0
3d	1LF4_A	Plasmepsin 2	N/D$_{214}$-O/L$_{301}$ 3.1<br>O/T$_{217}$-N/D$_{303}$ 2.8<br>O/S$_{218}$-CD/P$_{304}$<br>3.2 (2.3) 135°	O/D$_{214}$-CA/G$_{302}$<br>3.2 (2.2) 153°<br>O/G$_{302}$-N/F$_{305}$ 3.0	OD1/D$_{214}$-N/G$_{216}$<br>2.8<br>O/D$_{214}$-N/T$_{217}$ 3.5	
3e	2BJU_A	Plasmepsin 2	N/D$_{34}$-O/L$_{124}$ 2.9<br>O/D$_{34}$-N/L$_{126}$ 2.8	O/D$_{34}$-CA/G$_{125}$<br>3.4 (2.5) 142°	OD2/D$_{34}$-N/G$_{36}$ 2.8<br>O/D$_{34}$-N/S$_{37}$ 3.3	O/M$_{15}$-N/F$_{11}$ 2.8<br>N/Y$_{17}$-O/V$_{9}$ 2.9

			O/S_{37}-N/G_{127} 2.8			O/Y_{17}-N/V_{9} 2.9
3f	2BJU_A	Plasmepsin 2	N/D_{214}-O/L_{301} 3.1<br>O/T_{217}-N/D_{303} 2.8<br>O/S_{218}-CD/P_{304} 3.0 (2.2) 136°	O/D_{214}-CA/G_{302} 3.3 (2.3) 155°<br>O/G_{302}-N/F_{305} 2.9	OD1/D_{214}-N/G_{216} 3.0<br>O/D_{214}-N/T_{217} 3.4	
4a	3QVC_A, p	HAP zymogen	N/H_{32}-O/F_{121} 3.3<br>O/H_{32}-N/L_{123} 2.7<br>O/S_{35}-N/G_{124} 2.7	O/H_{32}-CA/G_{122} 3.5 (2.7) 138°	ND1/H_{32}-N/A_{34} 3.4<br>O/H_{32}-N/S_{35} 4.3	O/V_{12}-N/E_{86p} 3.0<br>N/S_{14}-O/N_{84p} 2.9<br>O/S_{14}-N/N_{84p} 2.9
4b	3QVC_A	HAP zymogen	N/D_{215}-O/L_{301} 3.0<br>O/T_{218}-N/D_{303} 2.9<br>O/S_{219}-CD/P_{304} 3.5 (2.6) 140°	O/D_{215}-CA/G_{302} 3.4 (2.3) 155°<br>O/G_{302}-N/F_{305} 3.0	OD1/D_{215}-N/A_{217} 3.0<br>O/D_{215}-N/T_{218} 3.5	
4e	3QVI_A, B	HAP protein	N/H_{32}-O/F_{121} 3.0<br>O/H_{32}-N/L_{123} 2.7<br>O/S_{35}-N/G_{124} 2.9	O/H_{32}-CA/G_{122} 3.7 (2.9) 131°	ND1/H_{32}-N/A_{34} 3.0<br>O/H_{32}-N/S_{35} 3.6	N/L_{13_A}-O/K_{7_B} 3.1<br>O/F_{15}-N/K_{7_B} 2.6
4f	3QVI_A	HAP protein	N/D_{215}-O/L_{301} 3.0<br>O/T_{218}-N/D_{303} 2.9<br>O/S_{219}-CD/P_{304} 3.7 (3.0) 124°	O/D_{215}-CA/G_{302} 3.4 (2.3) 155°<br>O/G_{302}-N/F_{305} 3.1	OD1/D_{215}-N/A_{217} 3.0<br>O/D_{215}-N/T_{218} 3.5	
5a	5N7N_A, p	Procathepsin D	N/D_{58}-O/L_{147} 3.1<br>O/D_{58}-N/L_{149} 2.8<br>O/S_{61}-N/A_{150} 2.8	O/D_{58}-CA/G_{148} 3.4 (2.5) 140°	OD1/D_{58}-N/G_{60} 2.9<br>O/D_{58}-N/S_{61} 3.4	O/V_{39}-N/F_{9p} 3.0<br>N/Y_{41}-O/T_{7p} 3.0<br>O/Y_{41}-N/T_{7p} 2.9
5b	5N7N_A	Procathepsin D	N/N_{249}-O/L_{336} 2.9<br>O/T_{252}-N/D_{338} 2.9<br>O/S_{253}-N/V_{339} 3.0	O/N_{249}-CA/G_{337} 3.5 (2.5) 169°<br>O/G_{337}-N/F_{340} 3.1	OD1/N_{249}-N/G_{251} 2.8<br>O/N_{249}-N/T_{252} 3.5	
4c	5N71_A	Cathepsin D	N/D_{58}-O/L_{147} 3.1<br>O/D_{58}-N/L_{149} 2.9<br>O/S_{61}-N/A_{150} 2.9	O/D_{58}-CA/G_{148} 3.4 (2.5) 143°	OD1/D_{58}-N/G_{60} 2.8<br>O/D_{58}-N/S_{61} 3.4	O/V_{39}-N/L_{35} 2.8<br>N/Y_{41}-O/V_{33} 3.1<br>O/Y_{41}-N/V_{33} 2.7
5d	5N71_A	Cathepsin D	N/N_{249}-O/L_{336} 2.9<br>O/T_{252}-N/D_{338} 2.9<br>O/S_{253}-N/V_{339} 3.1	O/N_{249}-CA/G_{337} 3.3 (2.3) 164°<br>O/G_{337}-N/F_{340} 2.9	OD1/N_{249}-N/G_{251} 2.8<br>O/N_{249}-N/T_{252} 3.5	
5e	5N7Q_A	Cathepsin D	N/D_{36}-O/L_{125} 2.9<br>O/D_{36}-N/L_{127} 2.8<br>O/S_{39}-N/A_{128} 2.8	O/D_{36}-CA/G_{126} 3.4 (2.4) 143°	OD1/D_{36}-N/G_{38} 3.0<br>O/D_{36}-N/S_{39} 3.4	O/V_{17}-N/L_{13} 3.0<br>N/Y_{19}-O/V_{11} 3.1<br>O/Y_{19}-N/V_{11} 2.8
5f	5N7Q_A	Cathepsin D	N/D_{227}-O/L_{314} 2.9<br>O/T_{230}-N/D_{316} 2.8<br>O/S_{231}-N/V_{317} 3.1	O/D_{227}-CA/G_{315} 3.3 (2.2) 159°<br>O/G_{315}-N/F_{318} 2.9	OD1/D_{227}-N/G_{229} 2.9<br>O/D_{227}-N/T_{230} 3.1	
6a	1MIQ_A, p	Proplasmepsin	N/D_{34}-O/L_{124} 3.1<br>O/D_{34}-N/L_{126} 2.5<br>O/S_{37}-N/G_{127} 2.7	O/D_{34}-CA/G_{125} 3.4 (2.5) 135°	OD1/D_{34}-N/G_{36} 3.3<br>O/D_{34}-N/S_{37} 3.8	O/I_{14}-N/E_{86p} 3.1<br>N/F_{16}-O/K_{84p} 2.9<br>O/F_{16}-N/K_{84p} 3.0
6b	1MIQ_A	Proplasmepsin	N/D_{214}-O/L_{301} 3.0	O/D_{214}-CA/G_{302}	OD2/D_{214}-N/G_{216}	

			O/T$_{217}$-N/D$_{303}$ 2.8<br>O/T$_{218}$-CD/P$_{304}$ 3.5 (2.6) 141°	3.3 (2.2) 156°<br>O/G$_{302}$-N/F$_{305}$ 3.2	2.8<br>O/D$_{214}$-N/T$_{217}$ 2.9	
6e	1QS8_A	Plasmepsin	N/D$_{34}$-O/L$_{124}$ 3.0<br>O/D$_{34}$-N/L$_{126}$ 2.9<br>O/S$_{37}$-N/G$_{127}$ 2.7	O/D$_{34}$-CA/G$_{125}$<br>3.5 (2.6) 142°	OD2/D$_{34}$-N/G$_{36}$ 3.0<br>O/D$_{34}$-N/S$_{37}$ 3.3	O/M$_{15}$-N/V$_{11}$ 2.9<br>N/Y$_{17}$-O/D$_{9}$ 3.2<br>O/Y$_{17}$-N/D$_{9}$ 2.9
6f	1QS8_A	Plasmepsin	N/D$_{214}$-O/L$_{301}$ 3.0<br>O/T$_{217}$-N/D$_{303}$ 2.7<br>O/T$_{218}$-CD/P$_{304}$ 3.7 (2.9) 127°	O/D$_{214}$-CA/G$_{302}$<br>3.3 (2.2) 157°<br>O/G$_{302}$-N/F$_{305}$ 3.2	OD2/D$_{214}$-N/G$_{216}$ 2.6<br>O/D$_{214}$-N/T$_{217}$ 3.3	
7a	5JOD_A, p	Proplasmepsin 4	N/D$_{34}$-O/L$_{124}$ 3.0<br>O/D$_{34}$-N/L$_{126}$ 2.7<br>O/S$_{37}$-N/G$_{127}$ 2.8	O/D$_{34}$-CA/G$_{125}$<br>3.5 (2.6) 140°	OD1/D$_{34}$-N/G$_{36}$ 2.9<br>O/D$_{34}$-N/S$_{37}$ 3.7	O/L$_{14}$-N/D$_{87p}$ 3.1<br>N/F$_{16}$-O/K$_{85p}$ 2.7<br>O/F$_{16}$-N/K$_{85p}$ 3.0
7b	5JOD_A	Proplasmepsin 4	N/D$_{214}$-O/L$_{301}$ 3.1<br>O/T$_{217}$-N/D$_{303}$ 2.8<br>O/S$_{218}$-CD/P$_{304}$ 3.4 (2.4) 151°	O/D$_{214}$-CA/G$_{302}$<br>3.2 (2.2) 158°<br>O/G$_{302}$-N/F$_{305}$ 3.0	OD1/D$_{214}$-N/G$_{216}$ 2.9<br>O/D$_{214}$-N/T$_{217}$ 3.2	
7e	1LS5_A	Plasmepsin 4	N/D$_{34}$-O/L$_{124}$ 3.1<br>O/D$_{34}$-N/L$_{126}$ 2.8<br>O/S$_{37}$-N/G$_{127}$ 3.2	O/D$_{34}$-CA/G$_{125}$<br>3.3 (2.5) 128°	OD1/D$_{34}$-N/G$_{36}$ 2.6<br>O/D$_{34}$-N/S$_{37}$ 4.3	O/M$_{15}$-N/V$_{11}$ 3.0<br>N/Y$_{17}$-O/D$_{9}$ 3.5<br>O/Y$_{17}$-N/D$_{9}$ 2.6
7f	1LS5_A	Plasmepsin 4	N/D$_{214}$-O/L$_{301}$ 2.8<br>O/T$_{217}$-N/D$_{303}$ 3.3<br>O/S$_{218}$-CD/P$_{304}$ 3.7 (2.9) 134°	O/D$_{214}$-CA/G$_{302}$<br>3.2 (2.2) 142°<br>O/G$_{302}$-N/F$_{305}$ 3.4	OD1/D$_{214}$-N/G$_{216}$ 2.5<br>O/D$_{214}$-N/T$_{217}$ 3.6	
8a	1QDM_A, p	Prophytepsin	N/D$_{36}$-O/L$_{126}$ 2.8<br>O/D$_{36}$-N/L$_{128}$ 2.7<br>O/S$_{39}$-N/G$_{129}$ 2.8	O/D$_{36}$-CA/G$_{127}$<br>3.5 (2.6) 139°	OD1/D$_{36}$-N/G$_{38}$ 3.1<br>O/D$_{36}$-N/S$_{39}$ 3.4	O/Q$_{17}$-N/R$_{13p}$ 2.7<br>N/F$_{19}$-O/K$_{11p}$ 2.8<br>O/F$_{19}$-N/K$_{11p}$ 2.8
8b	1QDM_A	Prophytepsin	N/D$_{223}$-O/L$_{313}$ 2.8<br>O/T$_{226}$-N/D$_{315}$ 2.8<br>O/S$_{227}$-N/V$_{316}$ 3.2	O/D$_{223}$-CA/G$_{314}$<br>2.9 (2.0) 135°<br>O/G$_{314}$-N/F$_{317}$ 2.7	OD1/D$_{223}$-N/G$_{225}$ 2.9<br>O/D$_{223}$-N/T$_{226}$ 3.9	
9a	1HTR_B, p	Progastricsin	N/D$_{32}$-O/M$_{121}$ 3.1<br>O/D$_{32}$-N/L$_{123}$ 2.9<br>O/S$_{35}$-N/A$_{124}$ 3.0	O/D$_{32}$-CA/G$_{122}$<br>3.4 (2.5) 140°	OD1/D$_{32}$-N/G$_{34}$ 2.9<br>O/D$_{32}$-N/S$_{35}$ 3.4	O/A$_{13}$-N/F$_{10p}$ 2.8<br>N/F$_{15}$-O/K$_{8p}$ 3.0<br>O/F$_{15}$-N/K$_{8p}$ 2.9
9b	1HTR_B	Progastricsin	N/D$_{217}$-O/L$_{304}$ 3.1<br>O/T$_{220}$-N/D$_{306}$ 2.9<br>O/S$_{221}$-N/V$_{307}$ 3.0	O/D$_{217}$-CA/G$_{305}$<br>3.5 (2.5) 165°<br>O/G$_{305}$-N/F$_{308}$ 2.8	OD1/D$_{217}$-N/G$_{219}$ 2.7<br>O/D$_{217}$-N/T$_{220}$ 3.4	
10a	1TZS_A, p	Procathepsin E	N/D$_{43}$-O/L$_{132}$ 3.0<br>O/D$_{43}$-N/L$_{134}$ 2.7<br>O/S$_{46}$-N/G$_{135}$ 2.8	O/D$_{43}$-CA/G$_{133}$<br>3.1 (2.2) 142°	OD1/D$_{43}$-N/G$_{45}$ 2.9<br>O/D$_{43}$-N/S$_{46}$ 3.4	N/F$_{26}$-O/R$_{9p}$ 2.8<br>O/F$_{26}$-N/R$_{9p}$ 3.0
10b	1TZS_A	Procathepsin E	N/D$_{228}$-O/L$_{317}$ 3.1<br>O/T$_{231}$-N/D$_{319}$ 2.8	O/D$_{228}$-CA/G$_{318}$<br>3.4 (2.3) 162°	OD1/D$_{228}$-N/G$_{230}$ 2.7	

		O/S$_{232}$-N/V$_{320}$ 3.2	O/G$_{318}$-N/F$_{321}$ 2.9	O/D$_{228}$-N/T$_{231}$ 3.1	
11c1T6E_X	Xylanase<br>inhibitor	N/D$_{29}$-O/A$_{141}$ 3.0<br>O/D$_{29}$-N/L$_{143}$ 2.8<br>O/G$_{32}$-N/A$_{144}$ 3.1	O/D$_{29}$-CA/G$_{142}$<br>3.5 (2.6) 141°	OD1/D$_{29}$-N/A$_{31}$ 3.2<br>O/D$_{29}$-N/G$_{32}$ 3.0	O/L$_{15}$-N/D$_{10}$ 2.7<br>N/T$_{17}$-O/T$_{8}$ 2.9<br>O/T$_{17}$-N/T$_{8}$ 2.9
11 1T6E_X<br>d	Xylanase<br>inhibitor	N/S$_{235}$-O/L$_{348}$ 2.9<br>O/L$_{238}$-N/G$_{350}$ 3.0<br>O/P$_{239}$-N/A$_{351}$ 3.1	O/S$_{235}$-CA/G$_{349}$<br>3.2 (2.2) 145°<br>O/G$_{349}$-N/Q$_{352}$ 3.2	OG/S$_{235}$-N/R$_{237}$ 3.0<br>O/S$_{235}$-N/L$_{238}$ 5.1	
11e1T6G_A	Xylanase<br>inhibitor	N/D$_{29}$-O/A$_{141}$ 3.0<br>O/D$_{29}$-N/L$_{143}$ 2.9<br>O/G$_{32}$-N/A$_{144}$ 3.0	O/D$_{29}$-CA/G$_{142}$<br>3.5 (2.6) 143°	OD1/D$_{29}$-N/A$_{31}$ 3.3<br>O/D$_{29}$-N/G$_{32}$ 3.0	O/L$_{15}$-N/D$_{10}$ 2.8<br>N/T$_{17}$-O/T$_{8}$ 2.9<br>O/T$_{17}$-N/T$_{8}$ 2.9
11f 1T6G_A	Xylanase<br>inhibitor	N/S$_{235}$-O/L$_{348}$ 2.9<br>O/L$_{238}$-N/G$_{350}$ 2.9<br>O/P$_{239}$-N/A$_{351}$ 3.1	O/S$_{235}$-CA/G$_{349}$<br>3.2 (2.2) 145°<br>O/G$_{349}$-N/Q$_{352}$ 3.1	OG/S$_{235}$-N/R$_{237}$ 3.2<br>O/S$_{235}$-N/L$_{238}$ 5.1	
12c3AUP_A	Basic 7S globulin	N/D$_{41}$-O/A$_{159}$ 3.1<br>O/D$_{41}$-N/L$_{161}$ 3.0<br>O/G$_{44}$-N/G$_{162}$ 2.9	O/D$_{41}$-CA/G$_{160}$<br>3.2 (2.2) 153°	OD1/D$_{41}$-N/N$_{43}$ 3.3<br>O/D$_{41}$-N/G$_{44}$ 2.9	O/L$_{22}$-N/D$_{17}$ 2.7<br>N/W$_{24}$-O/Q$_{15}$ 2.9<br>O/W$_{24}$-N/Q$_{15}$ 2.8
12 3AUP_A<br>d	Basic 7S globulin	N/S$_{265}$-O/L$_{361}$ 2.9<br>O/T$_{268}$-N/A$_{363}$ 2.9<br>O/P$_{269}$-N/R$_{364}$ 2.9	O/S$_{265}$-CA/G$_{362}$<br>3.3 (2.4) 135°<br>O/G$_{362}$-N/Q$_{365}$ 2.9	OG/S$_{265}$-N/S$_{267}$ 2.9<br>O/S$_{265}$-N/T$_{268}$ 4.9	
13c3VLA_A	EDGP<br>(Fragment)	N/D$_{40}$-O/A$_{155}$ 3.0<br>O/D$_{40}$-N/L$_{157}$ 2.9<br>O/G$_{43}$-N/G$_{158}$ 2.8	O/D$_{40}$-CA/G$_{156}$<br>3.4 (2.4) 149°	OD1/D$_{40}$-N/G$_{42}$ 3.4<br>O/D$_{40}$-N/G$_{43}$ 2.9	O/Q$_{21}$-N/D$_{16}$ 2.8<br>N/V$_{23}$-O/K$_{14}$ 2.9<br>O/V$_{23}$-N/K$_{14}$ 2.9
13 3VLA_A<br>d	EDGP<br>(Fragment)	N/S$_{271}$-O/I$_{374}$ 2.9<br>O/N$_{274}$-N/G$_{376}$ 2.9<br>O/P$_{275}$-N/H$_{377}$ 2.9	O/S$_{271}$-CA/G$_{375}$<br>3.6 (2.6) 147°<br>O/G$_{375}$-N/Q$_{378}$ 3.0	OG/S$_{271}$-N/I$_{273}$ 3.0<br>O/S$_{271}$-N/N$_{274}$ 4.7	
13e3VLB_A	EDGP<br>(Fragment)	N/D$_{40}$-O/A$_{155}$ 3.0<br>O/D$_{40}$-N/L$_{157}$ 3.2<br>O/G$_{43}$-N/G$_{158}$ 2.7	O/D$_{40}$-CA/G$_{156}$<br>3.6 (2.6) 151°	OD1/D$_{40}$-N/G$_{42}$ 3.7<br>O/D$_{40}$-N/G$_{43}$ 2.8	O/Q$_{21}$-N/D$_{16}$ 2.9<br>N/V$_{23}$-O/K$_{14}$ 2.8<br>O/V$_{23}$-N/K$_{14}$ 2.7
13f 3VLB_A	EDGP<br>(Fragment)	N/S$_{271}$-O/I$_{374}$ 3.1<br>O/N$_{274}$-N/G$_{376}$ 2.7<br>O/P$_{275}$-N/H$_{377}$ 2.9	O/S$_{271}$-CA/G$_{375}$<br>4.0 (3.2) 129°<br>O/G$_{375}$-N/Q$_{378}$ 3.4	OG/S$_{271}$-N/I$_{273}$ 3.5<br>O/S$_{271}$-N/N$_{274}$ 4.9	
Family: Retroviral protease (retropepsin)					
14c3IXO_A	HIV-1 protease	N/D$_{25}$-O/I$_{85}$ 3.0<br>O/A$_{28}$-N/R$_{87}$ 2.7<br>O/D$_{29}$-N/N$_{88}$ 3.1	O/D$_{25}$-CA/G$_{86}$<br>3.8 (2.7) 170°<br>O/G$_{86}$-N/M$_{89}$ 3.0	OD1/D$_{25}$-N/G$_{27}$ 2.9<br>O/D$_{25}$-N/A$_{28}$ 3.1	
14 3IXO_ B<br>d	HIV-1 protease	N/D$_{25}$-O/I$_{85}$ 3.0<br>O/A$_{28}$-N/R$_{87}$ 2.9<br>O/D$_{29}$-N/N$_{88}$ 3.4	O/D$_{25}$-CA/G$_{86}$<br>3.9 (2.9) 162°<br>O/G$_{86}$-N/M$_{89}$ 3.0	OD1/D$_{25}$-N/G$_{27}$ 2.8<br>O/D$_{25}$-N/A$_{28}$ 3.2	
14e5YOK_A	HIV-1 protease	N/D$_{25}$-O/I$_{85}$ 2.9<br>O/A$_{28}$-N/R$_{87}$ 2.8<br>O/D$_{29}$-N/N$_{88}$ 3.3	O/D$_{25}$-CA/G$_{86}$<br>3.7 (2.7) 167°<br>O/G$_{86}$-N/L$_{89}$ 3.1	OD1/D$_{25}$-N/G$_{27}$ 2.9<br>O/D$_{25}$-N/A$_{28}$ 3.2	

14f	5YOK_ B	HIV-1 protease	N/D_{25}-O/I_{85} 2.9<br>O/A_{28}-N/R_{87} 2.8<br>O/D_{29}-N/N_{88} 3.3	O/D_{25}-CA/G_{86} 3.7 (2.7) 170°<br>O/G_{86}-N/L_{89} 3.1	OD1/D_{25}-N/G_{27} 2.7<br>O/D_{25}-N/A_{28} 3.1
15c	3NR6_A	XMRV protease	N/D_{32}-O/L_{93} 2.9<br>O/A_{35}-N/R_{95} 2.8<br>O/Q_{36}-N/D_{96} 3.1	O/D_{32}-CA/G_{94} 3.5 (2.5) 163°<br>O/G_{94}-N/L_{97} 3.2	OD1/D_{32}-N/G_{34} 2.9<br>O/D_{32}-N/A_{35} 3.0
15 d	3NR6_B	XMRV protease	N/D_{32}-O/L_{93} 2.9<br>O/A_{35}-N/R_{95} 2.8<br>O/Q_{36}-N/D_{96} 3.1	O/D_{32}-CA/G_{94} 3.5 (2.5) 166°<br>O/G_{94}-N/L_{97} 3.2	OD1/D_{32}-N/G_{34} 3.0<br>O/D_{32}-N/A_{35} 3.1
15e	3SLZ_A	XMRV protease	N/D_{32}-O/L_{93} 2.8<br>O/A_{35}-N/R_{95} 2.9<br>O/Q_{36}-N/D_{96} 3.7	O/D_{32}-CA/G_{94} 3.4 (2.3) 168°<br>O/G_{94}-N/L_{97} 3.1	OD1/D_{32}-N/G_{34} 3.0<br>O/D_{32}-N/A_{35} 3.1
15f	3SLZ_B	XMRV protease	N/D_{32}-O/L_{93} 2.8<br>O/A_{35}-N/R_{95} 2.9<br>O/Q_{36}-N/D_{96} 3.8	O/D_{32}-CA/G_{94} 3.4 (2.4) 166°<br>O/G_{94}-N/L_{97} 3.1	OD1/D_{32}-N/G_{34} 2.9<br>O/D_{32}-N/A_{35} 3.1
			Family: Dimeric aspartyl proteases		
16c	4Z2Z_A	DDI1 protease	N/D_{220}-O/I_{289} 2.9<br>O/A_{223}-N/L_{291} 2.8<br>O/Q_{224}-N/D_{292} 3.1	O/D_{220}-CA/G_{290} 3.6 (2.6) 166°<br>O/G_{290}-N/M_{293} 3.3	OD1/D_{220}-N/G_{222} 2.9<br>O/D_{220}-N/A_{223} 3.2
16 d	4Z2Z_B	DDI1 protease	N/D_{220}-O/I_{289} 2.9<br>O/A_{223}-N/L_{291} 2.9<br>O/Q_{224}-CB/D_{292} 3.9 (3.1) 136°	O/D_{220}-CA/G_{290} 3.5 (2.5) 154°<br>O/G_{290}-N/M_{293} 3.3	OD1/D_{220}-N/G_{222} 2.9<br>O/D_{220}-N/A_{223} 3.2
17c	5C9F_A	ApRick protease	N/D_{140}-O/L_{209} 3.0<br>O/A_{143}-N/M_{211} 2.9<br>O/S_{144}-N/S_{212} 2.9	O/D_{140}-CA/G_{210} 3.4 (2.3) 162°<br>O/G_{210}-N/L_{213} 3.4	OD1/D_{140}-N/G_{142} 3.1<br>O/D_{140}-N/A_{143} 3.1
			Family: LPG0085-like		
18c	2PMA_A	Protein Lpg0085	N/D_{47}-O/L_{145} 2.9<br>O/A_{50}-N/R_{147} 2.8<br>O/K_{51}-N/D_{148} 3.1	O/D_{47}-CA/G_{146} 3.5 (2.5) 154°<br>O/G_{146}-N/A_{149} 3.2	OD1/D_{47}-N/G_{49} 3.0<br>O/D_{47}-N/A_{50} 3.4
18 d	2PMA_I	Protein Lpg0085	N/D_{47}-O/L_{145} 2.8<br>O/A_{50}-N/R_{147} 2.8<br>O/K_{51}-N/D_{148} 3.1	O/D_{146}-CA/G_{146} 3.4 (2.4) 158°<br>O/G_{146}-N/A_{149} 3.2	OD1/D_{47}-N/G_{49} 2.9<br>O/D_{47}-N/A_{50} 3.4

Table S3. Conserved geometric parameters (distance and angle) of contacts between hydrolase and ligand in 9 acid proteases pepsin-like and retroviral protease (retropepsin) families.

N	PDB ID	Protein	D-loops / ligand		
			Superfamily: Acid proteases		
			Family: Pepsin-like		
1e	6XCZ_A	Pepsin	OD1/D$_{32}$-O2/ROC$_{401}$ 2.4	O/G$_{34}$-N2/ROC$_{401}$ 3.0	N/S$_{36}$-HOH$_{527}$ 2.8<br>HOH$_{527}$-OD1/ROC$_{401}$ 2.6
1f	6XCZ_A	Pepsin	OD2/D$_{215}$-O2/ROC$_{401}$ 2.8	O/G$_{217}$-C21/ROC$_{401}$ 3.3 (2.4) 141°<br>O/G$_{217}$-CM/ROC$_{401}$ 3.3 (2.5) 131°	N/S$_{219}$-HOH$_{645}$ 3.3<br>HOH$_{645}$-N3/ROC$_{401}$ 3.2
2e	3K1W_A	Renin	OD1/D$_{38}$-N27/BFX$_{342}$ 3.0	O/G$_{40}$-C24/BFX$_{342}$ 3.5 (2.5) 148°	OG/S$_{41}$-C17/BFX$_{342}$ 3.9 (2.9) 160°
2f	3K1W_A	Renin	OD2/D$_{226}$-N27/BFX$_{342}$ 2.7	O/G$_{228}$-C34/BFX$_{342}$ 3.2 (2.3) 135°	OG/S$_{230}$-C37/BFX$_{342}$ 3.5 (2.9) 117°
3e	2BJU_A	Plasmepsin 2	OD1/D$_{34}$-C5/IH4$_{1330}$ 3.9 (2.8) 159°	O/G$_{36}$-C6/IH4$_{1330}$ 3.9 (3.1) 134°	N/A
3f	2BJU_A	Plasmepsin 2	OD2/D$_{214}$-N13/IH4$_{1330}$ 2.6	O/G$_{216}$-C32/IH4$_{1330}$ 3.2 (2.8) 106°<br>O/G$_{216}$-C22/IH4$_{1330}$ 3.9 (2.9) 161°	N/S$_{218}$-O1/IH4$_{1330}$ 2.9
4e	3QVI_A	HAP protein	NE2/H$_{32}$-HOH$_{338}$ 3.1<br>HOH$_{338}$-CBB/ K95$_{329}$ 3.4 (2.6) 129°	O/A$_{34}$-OAI/K95$_{329}$ 2.9	OG/S$_{35}$-OAG/K95$_{329}$ 3.0
4f	3QVI_A, B	HAP protein	OD2/D$_{215_A}$-HOH$_{364}$ 2.5<br>HOH$_{364}$-OAI/ K95$_{329}$ 2.9	OE2/E$_{278A_B}$-NBD/K95$_{329}$ 2.7	OE2/E$_{278A_B}$-NBD/K95$_{329}$ 2.7
5e	5N7Q_A	Cathepsin D	OD1/D$_{36}$-OH/STA$_{504}$ 2.6	O/G$_{38}$-N/ALA$_{505}$ 2.9	N/A$_{40}$-HOH$_{735}$ 2.8<br>HOH$_{735}$-CB/ALA$_{505}$ 3.9 (3.5) 104°
5f	5N7Q_A	Cathepsin D	OD2/D$_{227}$-OH/STA$_{504}$ 2.6	O/G$_{229}$-N/STA$_{504}$ 3.1	N/S$_{231}$-O/VAL$_{502}$ 2.9
6e	1QS8_A	Plasmepsin	OD2/D$_{34}$-OH/STA$_{404}$ 2.5	O/G$_{36}$-N/ALA$_{405}$ 3.1	N/A$_{38}$-HOH$_{349}$ 2.8<br>HOH$_{349}$-CB/ALA$_{405}$ 3.9 (3.5) 103°
6f	1QS8_A	Plasmepsin	OD1/D$_{214}$-OH/STA$_{404}$ 2.4	O/G$_{216}$-N/STA$_{404}$ 3.5	N/T$_{218}$-O/VAL$_{402}$ 3.0
7e	1LS5_A	Plasmepsin 4	OD1/D$_{34}$-OH/STA$_{4}$ 2.3	O/G$_{36}$-N/ALA$_{5}$ 2.9	CB/S$_{37}$-CB/ALA$_{5}$ 3.6
7f	1LS5_A	Plasmepsin 4	OD1/D$_{214}$-OH/STA$_{4}$ 3.1	O/G$_{216}$-N/STA$_{4}$ 2.7	N/S$_{218}$-O/VAL$_{2}$ 2.8
			Family: Retroviral protease (retropepsin)		
14f	5YOK_ A	HIV-1 protease	OD1/D$_{25}$-O2/8Z0$_{101}$ 2.9	O/G$_{27}$-HOH$_{308}$ 3.0<br>HOH$_{308}$-N5/8Z0$_{101}$ 2.9	N/D$_{29}$-HOH$_{308}$ 2.9<br>HOH$_{308}$-N5/8Z0$_{101}$ 2.9

14f	5YOK_B	HIV-1 protease	OD1/D_{25}-O2/8Z0_{101} 2.6	O/G_{27}-N3/8Z0_{101} 3.3	N/D_{29}-O3/8Z0_{101} 3.0
15e	3SLZ_A	XMRV protease	OD1/D_{32}-O51/$3TL_{126}$ 2.5	O/G_{34}-N1/$3TL_{126}$ 2.9	N/Q_{36}-O4/$3TL_{126}$ 2.9<br>OE1/Q_{36}-N4/$3TL_{126}$ 3.1
15f	3SLZ_B	XMRV protease	OD1/D_{32}-O51/$3TL_{126}$ 2.8	O/G_{34}-N51/$3TL_{126}$ 3.0	N/Q_{36}-O54/$3TL_{126}$ 3.0<br>OE1/Q_{36}-N54/$3TL_{126}$ 3.1
